# Supplementary material for: Societal benefits of halving agricultural ammonia emissions in China far exceed the abatement costs
Source: Nat Commun. 2020 Aug 31;11:4357. doi: 10.1038/s41467-020-18196-z (PMC7459339; doi:10.1038/s41467-020-18196-z)
Supplement: Supplementary file 1 — Supplementary Information [file 41467_2020_18196_MOESM1_ESM.pdf]

Supplementary information for

Societal benefits of halving agricultural ammonia emissions in China far exceed the abatement costs

By Zhang et al.

Supplementary figures

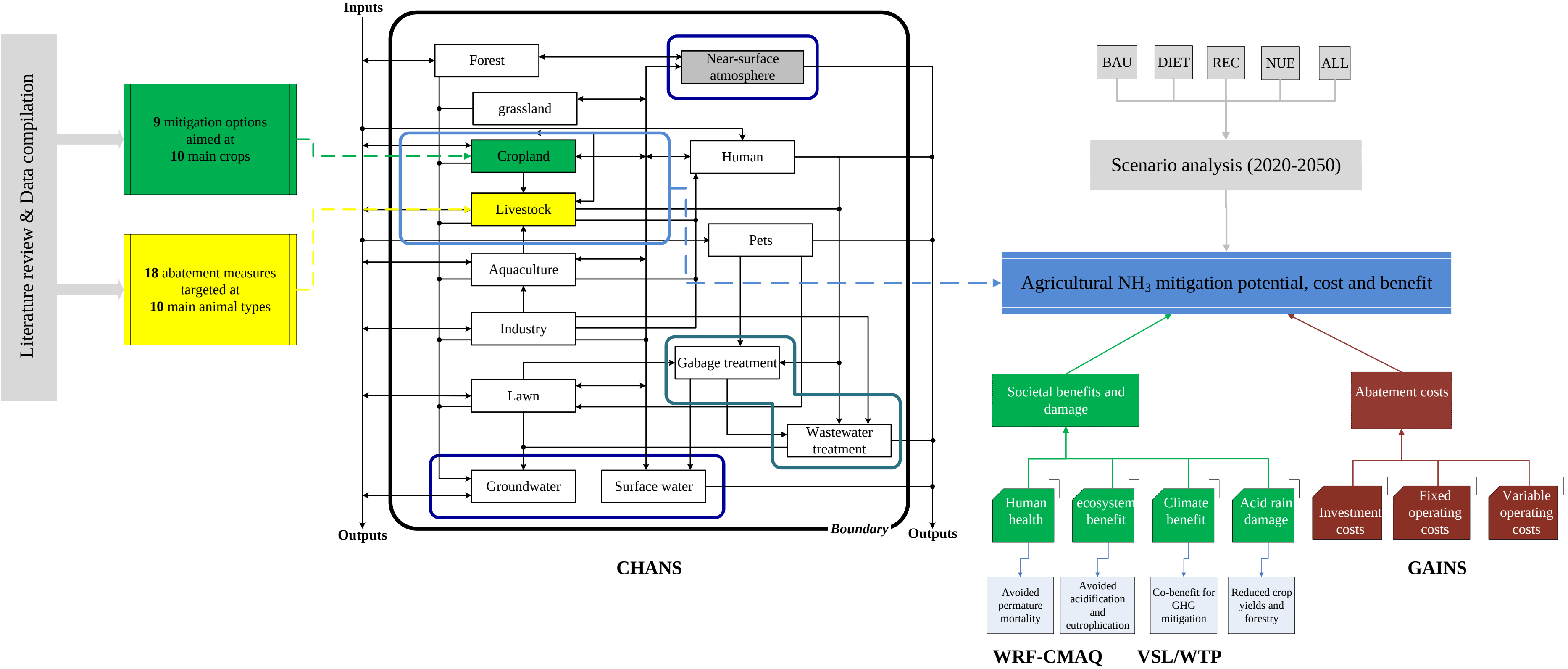

Supplementary Figure 1 Integrated assessment framework of agricultural NH_3 mitigation. Agricultural NH_3 emissions and mitigation potential in China is first assessed by CHANS model. The abatement costs and benefits under different scenarios are then evaluated with a systematic combination of WRF-CMAQ model, GAINS model and VSL methods.

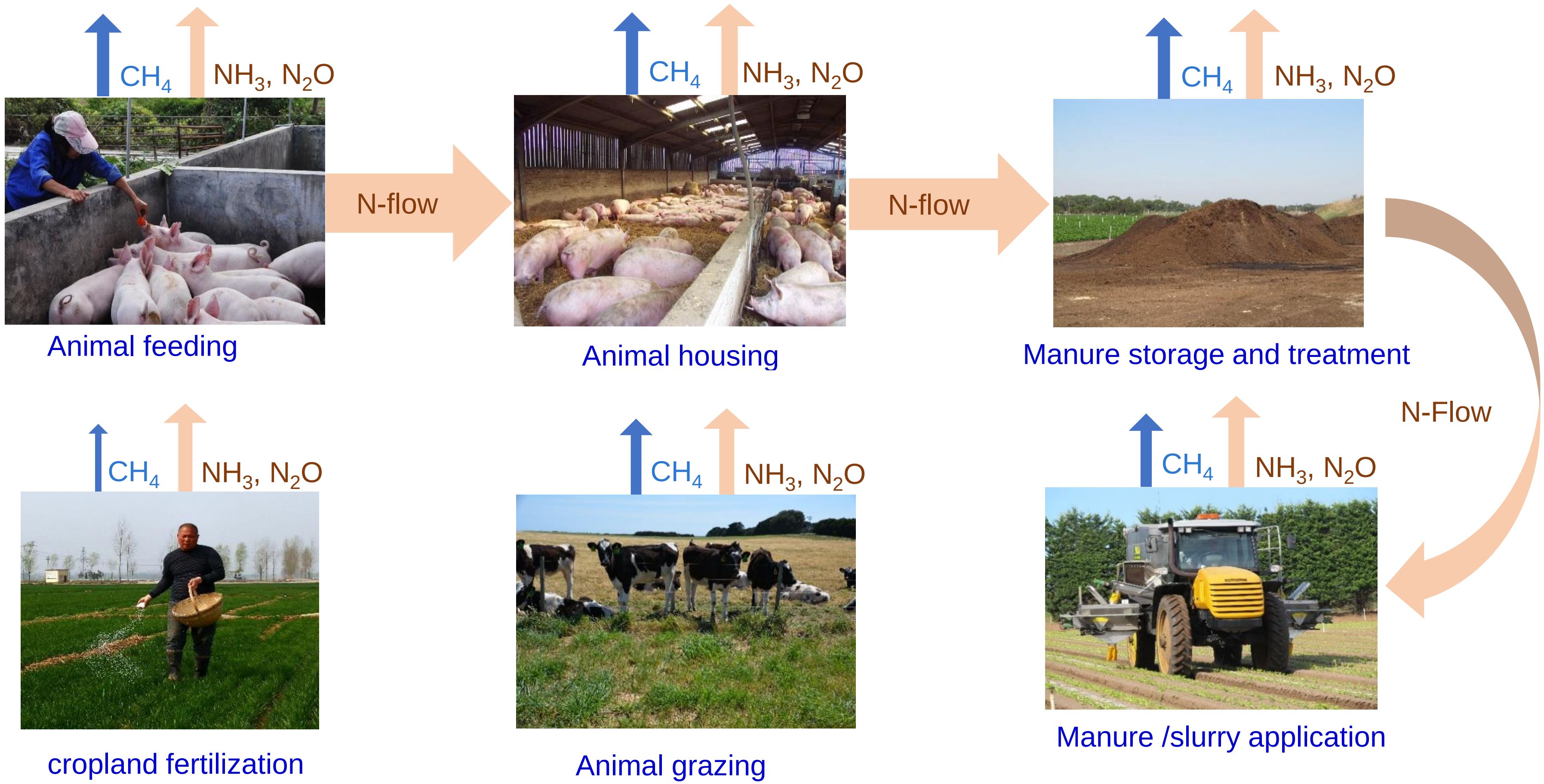

Supplementary Figure 2 NH_3 and GHG emissions from agricultural production. This figure demonstrates the possible co/side effects of NH_3 reduction on N_2O and CH_4 emission.

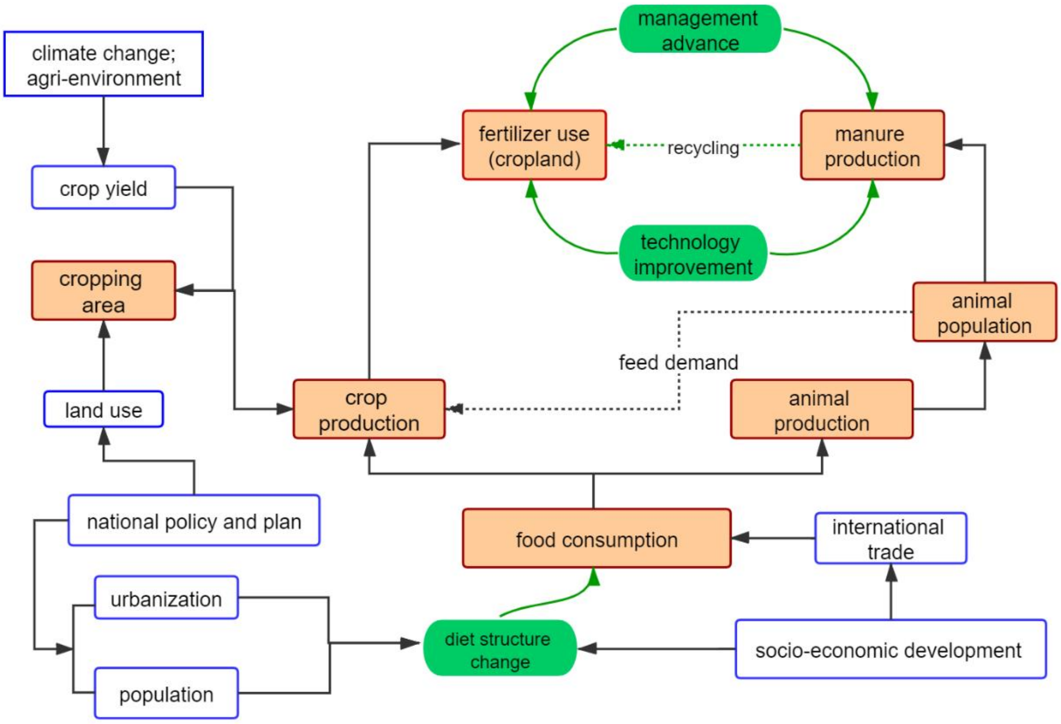

Supplementary Figure 3 The framework of input drivers in scenario analysis. White rectangles with blue edges represent the main input parameters, black arrows stand for data flow, green rounded rectangles represent different scenarios, while orange rounded rectangles represent the corresponding activity data output.

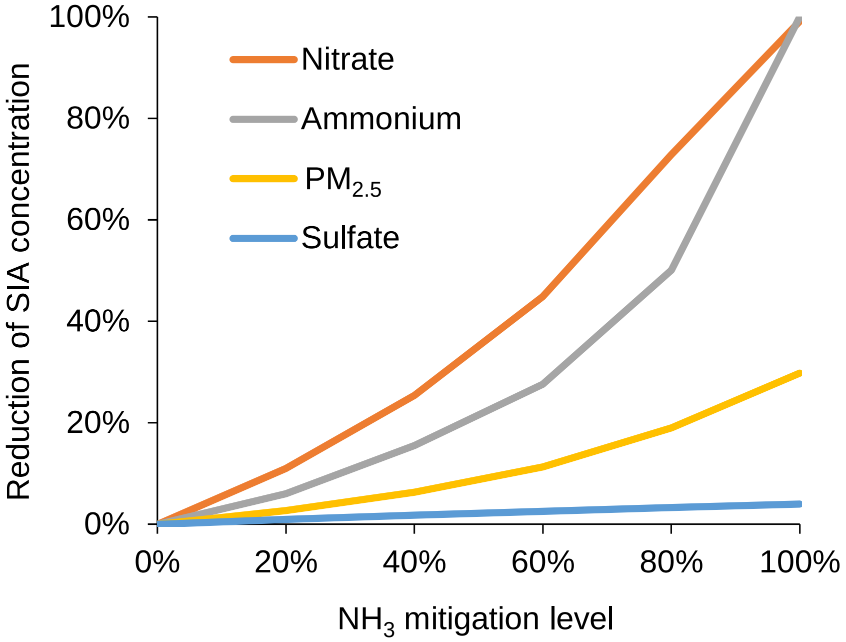

Supplementary Figure 4 Responses of annual concentration of $PM_{2.5}$ compositions to NH_3 mitigation in China simulated by WRF-CMAQ. Original data is derived from Xu et al. (2017)[1].

Supplementary Table 1 List of mitigation options available for cropland in China

Code	approach	Target crops	Abatement efficacy	Reference
C1	Right source-urea substitution (US)	all crops, vegetable, fruits	20-75%	[2-4]
C2	Right source- use of enhanced efficiency N fertilizer (EENF)	all crops, vegetable, fruits	57-68%	[3-5]
C3	Right source-Application of organic fertilizer (OF)	rice, wheat, maize, vegetable, fruits	26-50%	[6, 7]
C4	Right rate-optimizing N rate (OPR)	all crops, vegetable, fruits	48-65%	[2, 4, 8]
C5	Reducing basal N fertilizer proportion (RBF)	rice, wheat, maize, vegetable, fruits	26 -62%	[9, 10]
C6	Split fertilization (SF)	wheat, maize, rice, vegetable, fruits	14 -52%	[4, 11]
C7	Deep placement of N fertilizer (DP)	rice, wheat, maize, cotton, fruits	27-87%	[2, 4]
C8	Irrigation (IR)-fertilizer and water best management	rice, wheat, maize, vegetable, cotton	40-70%	[2, 12]
C9	Soil amendment (AM)	rice, wheat, maize, vegetable, fruits	31-54%	[11, 13]

Note: Chinese specific data are preferred to improve the accuracy of assessment when many studies are available. When several studies are referenced, the range of abatement efficacy refers to mean values provided in the studies (meta-analysis results).

Supplementary Table 2 List of mitigation options available for livestock production in China

Code	Approach	Target animals/Applicability	Abatement efficacy	Reference
**Feeding**				
L1	low crude protein (LCP) feeding	Indoor-pig, poultry, cattle	10-20%	[2, 14]
L2	dietary additives (DA)	Indoor-pig, cattle, poultry	33-45%	[14, 15]
L3	phrase feeding (PF) strategy	Intensive livestock farming – pig, poultry(indoor)	~10%	[14, 16-18]
**Housing**				
L4	floor adaption (FAD)	Indoor-pig, cattle, poultry	10-50%	[14, 16]
L5	bedding materials (BED)	Indoor-pig, cattle	20-50%	[14, 19]
L6	air scrubbing techniques (AST) or bio filter	Indoor-pig, poultry	70-95%	[20, 21]
L7	frequent manure removal (FMR)	Indoor-pig, poultry, cattle	25-30%	[14, 22]
L8	rapid manure drying (RMD)	solid manure (pig, poultry)	70-90%	[20, 22]
**Storage and processing**				
L9	solid-liquid separation (SLS)	all kinds of manure	20-30%	[23, 24]
L10	improvement on storage facility (ISF)	all kinds of manure	20-30%	[14, 22]
L11	manure surface covers (SC)	all kinds of manure	40-60%	[2, 22]
L12	Acidification by additives (ADD)	slurry (cattle, pig, poultry)	18-70%	[25, 26]
L13	composting (COM) (aeration, turning, compaction)	Solid manure (cattle, pig, horse)	~55%	[27]
**Application**				
L14	band spreading (BS)	slurry	38-75%	[2, 28]
L15	incorporation (INC)	Solid manure	45-65%	[14, 29]
L16	injection (slurry only) (INJ)	liquid manure-slurry	80-90%	[22, 30]
L17	Irrigation (IR)	slurry dilution via irrigation	~30%	[3, 14]
**Grazing**				
L18	adjust the grazing time (AGT)	cattle, sheep, goat, horse	~10%	[31]

Note: Chinese specific data are preferred to improve the accuracy of assessment when many studies are available. When several studies are referenced, the range of abatement efficacy refers to mean values provided in the studies (meta-analysis results).

Supplementary Table 3 Practical applicability and cost localization of NH_3 mitigation options applied to cropland system

Code	Abbr.	Details	Cost	Ref
C1	US	partly substitute urea-based fertilizer with other N fertilizer, e.g. ammonium sulphate and ammonium nitrate, urea phosphate, calcium ammonium nitrate, monoammonium phosphate, and diammonium phosphate could significantly reduce NH_3 emission by 88.3, 82.9, 76.2, 67.3, 51.9, and 51.2%, respectively, compared with the use of urea.[2]	urea: 1000 CNY/t. Use calcium ammonium nitrate (total nutrient≥34 %, total N=26%) to represent nitrate-based fertilizers, average price:1400 CNY/t	[3, 32-34]
C2	EENF	EENF include the urease inhibitors (UI, e.g. NPBT), nitrification inhibitors (NI, e.g. DCD), control release fertilizer (CRF, e.g. polymer-coated and sulfur-coated urea), EENF could increase yield and NUE by synchronizing N release and demand as well as to minimize N losses.	CRF: 2500 CNY/t, NBPT:150CNY/kg, DCD:8CNY/kg China's total production of EENF was 21 million tons by 2015, with a total promotion area of 33 million hectares.	[34, 35]
C3	OF	increase the recycling of organic wastes to substitute synthetic fertilizer. organic fertilizers are incorporated into soil as basal fertilizers, the proportion of organic substitution for chemical fertilizer varies in different crops and regions, typically organic manure as basal fertilizer providing 30% of N nutrients to crops and 50% to fruits and vegetables. N content in typical organic manure fertilizers stands at 1.2%.	average market price for organic fertilizer :1100 CNY/t,	[33, 36, 37]
C4	OPR	this measure calls for a direct reduction in N fertilizer use for certain crops. The optimal N rate was 28% lower than the traditional N rate of the studies included in meta-analysis.	Reductions in crop-specific N fertilizer rates are based on recommended rates for main crops (see supplementary Table 14) we assume no additional cost for OPR. the fertilizer utilization efficiency is assumed to increase to more than 40%.	[4]
C5	RBF	the minimum percentage of BF reduction is 10% to avoid over basal N fertilization and increased N uptake along crops growth, thus reducing NH_3 emission, N leaching, and runoff.	Assume 10% more labour input is required. Minimum labour cost :17CNY/hour	[22, 38, 39]
C6	SF	compared to single application, split the total amount of N fertilizer into 3-4 applications for basal fertilization and top dressing, and shift from mid-season drainage to intermittent irrigation	more labour input is required for corn, wheat, rice, fruits split fertilization. Minimum labour cost :17CNY/hour	[4, 37]
C7	DP	deep placement significantly decreased floodwater NH_4^+-N concentration and NH_3 volatilization compared to surface application, the minimum depth of the deep placement of fertilizer N was 5 cm below the soil surface, usually supplied at 20cm depth from soil surface below plants.	Increased machine investment and operation costs for maize/cotton/fruits deep fertilization. Average fertilizer applicator price: 22000 CNY per machine.	[36, 40]
C8	IR	Irrigation with at least 5 mm water immediately following fertilizer application has been shown to reduce NH_3 emission by up to 70%. The irrigation amount and time should be reasonably determined to achieve the integration of water and fertilizer management.	High-efficient irrigation systems allow for labour and water savings in vegetable, fruits and cotton fields. Subsurface drip irrigation system costs include 15000 CNY/ha initial investment and installation cost (lifespan=10 years) and annual maintenance and renewal cost of smaller diameter polytube at 1500 CNY/ha (10%),	[16, 41, 42]

			while labour and pesticides (cotton) and irrigation costs will be saved.	
C9	AM	Apply soil amendment like biochar, lime, compost tea, organic acid to provide soil fertility improvements and increase N uptake by plants	More labour input is required. Cost of soil amendment is 600~1800 CNY/ha. Here we use biochar (2900 CNY/ tonne) to represent all kinds of amendment.	37

Note: CNY means Chinese Yuan, 2015 price.

Supplementary Table 4 Practical applicability and cost localization of NH_3 mitigation options applied to livestock operations

Code	Abbr.	Details	Cost	Ref
**Feeding**			**Additional cost for feed and labour**	
L1	LCP	LCP is a method of dietary change, a lower protein (nitrogen) content of animal feeds leads to reduced N excretion. This will basically affect all later stages, although the effect on stage 4, grazing, may be different)	The cost of low-protein animal feeding is relatively low. Here LCP is regarded as a cost-effective option in NH_3 emission control. This option can yield additional benefit since China wishes to put more low-protein feed in animal farms in order to reduce soybean imports.	15, 43-45
L2	DA	Dietary additives like yucca extract for ruminal animal; non-starch polysaccharides (NSP) for piggery; corn-dried distiller grain with soluble (DDGS) for laying hen； mineral zeolite; probiotics. These options mainly applicable to confined livestock because grazing animal's feed mainly come from grassland and excretion being directly returned to grassland.	Dietary additives costs need to be assessed of market prices to provide benefits not only to the environment, but also to animal welfare and producer, abundant solid evidence from scientific trials and practical experience indicate it is cost-effective.	45, 46
L3	PF	3 or 4 phase-feeding for intensive livestock farming could decrease N excretion and nitrogen volatilization losses	additional costs of phase-feeding are from labour and housing facility adjustment, economic analysis indicated that phase-feeding may lower the cost of feed consumed	22
**Housing**			**Additional operating cost for material, gas, electricity and labour**	
L4	FA	Applicable mainly for intensive rearing of poultry and pigs, adaptions like adjusting floor type to slotted floor	options that prevent NH_3 emission from animal housing basically focus on reducing the surface area and exposure time of manure in the animal house, for example, flushing systems or other means of immediate transport of manure into storage, installing filtration or biofiltration, or bio-fermented mattress materials that meet the requirements of animal epidemic prevention.	14, 29
L5	BED	Agricultural wastes (such as wheat husk, rice husk, rice chaff, straw, sawdust, ash soil, etc.) can be used as bedding materials		47
L6	AST	establishment of air cleaning infrastructures like biofilters and acid scrubber systems. removal efficiencies of NH_3 by acid scrubbers depending on their pH-set values, more practical and effective for large scale operations		17
L7	FMR	manure quick removal from a stable floor to a closed storage system by belts transport, scrape, or flush	It is generally cost-effective though L7 could introduce investment cost, e.g. manure conveyor belt technique, it can help farmers efficiently complete the manure cleaning work in the farm to save the labour cost.	14, 29
L8	RMD	dry the manure insider or outside the house using manure drying system to over 80% dry matter content reduces NH_3 emission.	Compared with manure flushing method, RMD only needs a quarter of the volume of the flushing tank, which can greatly reduce the construction cost of the collecting tank and water cost though extra thermal drying-energy cost	
**Processing and storage**			**Additional operating cost for material, energy and labour**	
L9	SLS	separation of solid and liquid manure by slatted floor		14, 29, 48

L10	ISF	Include outdoor tanks made of concrete or steel or use concrete, corrugated iron or polyester caps	cost of manure processing and storage mainly includes one-time input cost of equipment and normal operation. The equipment investment cost includes the construction cost of solid-liquid separation equipment, anaerobic fermentation tank, biogas slurry storage tank and other fixed equipment, as well as the land rent. Variable costs include labour cost, raw material cost, transportation cost, equipment maintenance cost, water and electricity cost, etc. Meantime, improved manure storage as a NH_3 abatement strategy could reduce the cost to cover the storage and reduced rainwater accumulation in uncovered stores. The costs for manure storage techniques refer to storage capacities of a volume of 500 m^3. The abatement costs per farm type ranged from 18-124 CNY for floating plastic covers and from 16-85 CNY for concrete covers	
L11	SC	The most suitable surface cover depends on the forms of manure: solid manure storages via compaction and covering while liquid manure storage via acidification and covers of straw or artificial films. Floating covers like natural crust or plastic film, straw as economical method for lagoons, structural covers are suited to concrete slurry tanks.		
L12	ADD	acidic additives or Cationic adsorbent to lower the manure pH. (Acidification)		
L13	ACP	Measures include negative pressure aeration, turning, compaction during composting.		
**Application**			**Additional facilities and operating cost for labour, fuel**	
L14	BS	Band application followed by covering soil is a relatively cost-effective method	Manure transportation to the field and land spreading rely on availability of suitable labour and machinery. The additional costs of manure recycling techniques are related to the volumes of manure to be spread, farms size, fuel cost, labour costs and in depreciation and interest rates. Detailed cost analysis could be found in Reis et al. (2015)[14]	14, 17, 29, 49
L15	INC	immediate incorporation by ploughing (within 4 h after application)		
L16	INJ	deep and shallow injection of liquid manure and immediate incorporation by ploughing (within 12 h after application) of solid manure.		
L17	IR	Irrigation immediately following manure field application could reduce NH_3 emission by up to 70% because the irrigation water washes the N into the soil allowing NH_4^+ to be adsorbed on cation exchange sites and reducing the amount of NH4+ available for NH_3 emission.		
**Grazing**				
L18	AGT	adjust the grazing time for grazing animals according season: shorter grazing period in summer, prolong the grazing time in winter. During grazing, less NH_3 was emitted because manure, especially the urine from grazing animals often bind relatively quickly with the soil and do not volatilize as much as in confined operations.	Assume only little additional labour cost generated since free grazing on pasture is most common in Chinese grassland systems. Extend the grazing time for grazing animals. Cost savings based on the reduced need for building floor scraping and slurry handling, together with reduced silage production costs	31

Supplementary Table 5 Current situation of Chinese farming practice and optimal practice assumption

Code	Abbr.	Historical practice or current adoption	Optimal practice or Maximum feasible adoption rate	References
C1	US	urea-based fertilizer is the most common form of fertilizer nitrogen used in China, accounting for average 197 kg N ha^{-1} yr^{-1} application rate compared to 74 kg N ha^{-1} yr^{-1} worldwide	replacement of urea-based fertilizer with nitrate-based fertilizer	[37]
C2	EENF	Limited use of EENF in China. Currently urease inhibitors are rarely used by Chinese farmers since they prefer other options that may provide similar benefits without incurring the additional cost of these products.	CRF, NBPT; DCD (reduce N application rate by applying EENFs without loss of yield, in general, UI and NI is applied at rates equivalent to 0.025% and 5% of N nutrient (w/w)). For wheat, apply urea amended with 0.12% (w/w) Limus (a new urease inhibitor consisting of 75% N-(n-butyl) NBPT and 25% N-(n-propyl) NPPT	
C3	OF	only 30% of livestock manure N is recycled back to agricultural land in China[50] while in European Union this share is more than 65%[51].	60% of manure N being efficiently recycling to croplands. In total organic fertilizer will account for 50% N input to cropping system. Apply to 30% rice, 30% wheat, 50% maize, 50% vegetables and 60% fruits fields	[52] [32]
C4	OPR	20-30% Over-application of chemical N fertilizer to cropland. Farmers are reluctant to adopt the OPR due to concerns of yield loss.	Reduce chemical fertilizer N input by 22% for rice, 24% for wheat, 18% for maize, 38% for cotton, 24% for vegetables and 11% for fruits.	[53]
C5	RBF	Farmers are used to applying large amounts of basal fertilizers at the early stage	Assume 100% application to rice, wheat, maize, vegetable and fruits by 2050 with respective optimal proportion of basal fertilizer.	[4, 32]
C6	SF	<40%. Nitrogen fertilizer applied in two or three times can reduce the overall amount of fertilizer applied and improve NUE.	Assume 100% adaption of increased splitting fertilization	[32]
C7	DP	<30%. the traditional artificial fertilization method (spreading and surface fertilization) still dominates, the mechanical fertilization only accounts for about 30% of the planting area of main crops.	Assume 80% adoption of deep placement of fertilizer for main crop types. The machinery level will be up to 70%.	National Agriculture Mechanization Extension Plan (2011-2015) [54]

C8	IR	<50 %, the irrigated area in China has increased to 66 million ha in 2015, and the area under water-saving irrigation techniques has increased to 28 million ha in 2015	Improved irrigation facilities and methods to obtain maximum NH_3 mitigation and crop yield. high-efficiency irrigations shall be installed on 22.5 Mha croplands (20 Mha new areas). Effective utilization coefficient of irrigation in farmland is up to 0.6 in 2030 and 0.8 in 2050.	National Agricultural Water-Saving Outline (2012-2020) [55]
C9	AM	although there are many kinds of soil amendments products developed, their promotion and application has been limited due to the high cost. Many farmers do not know how to use due to lack of proper publicity and technical training	Assume 50% adoption rate of soil amendment for polluted cropland.	[56, 57]
L1	LCP	Limited application.	It is appropriate only for medium and large-scale farms. Assume 50% livestock population are fed with LCP.	Expert opinion
L2	DA	Limited application, most common for pig, poultry, beef and cow but not applied to for goat farms	50% of pig and poultry, 30% of beef and dairy cattle, 20% of sheep and goat	Expert opinion
L3	PF	Limited, mainly appliable for medium and large-scale farms.	Assume 100% of livestock from medium and large-scale farms will be reared with phase-feeding	Expert opinion
L4	FA	Limited. For those farms that not suitable to adopt beddings, it is advisable to employ slatted floor. Mainly apply for medium and large-scale pig and poultry farms.	Assume 100% implementation rate for large-scale farms.	[47] Expert opinion
L5	BED	Limited application because traditional bedding requires large unit area, and labor input. Advanced bedding would introduce 30-40% higher cost.	Assume 80% for piggery and 50% for cattle in medium and large-scale farms.	Expert opinion Authors' assumption
L6	AST	Limited. the overall mechanization rate of animal husbandry is less than 20%	Assume 50% for piggery and 60% for poultry. By 2025 and 2050, the overall mechanization rate of animal husbandry should reach about 50% and 100%.	
L7	MFR	Limited application. By 2015, a total of 61,000 large-scale breeding farms (districts) have installed waste treatment and recycling facilities.	Assume 90% large-scale livestock and poultry farms adopt frequent manure removal technology.	[47, 58]
L8	RMD	Limited application.	Assume 50% for piggery and 60% for poultry	
L9	SLS	<40%. The solid and liquid manure in the feedlot are not separated. In north China, the faeces from manual cleaning are directly stacked outdoors without any treatment. Most of the liquid manure is discharged into nearby rivers.	Assume 80% effective solid-liquid manure separation	[28, 59]

L10	ISF	Currently, intensive animal feeding operations allow 45% of manure generated to be discharged into streams/rivers.	Assume 60% new-built storage facility and 100% improvement on current facility	
L11	SC	nearly 20% is left unmanaged. For treated manure, 35% is being stored, while about 55% adopt anaerobic method for future utilization, and about 2% adopt the biological fermentation bed.	Assume 80% implementation rate of effective surface cover during manure storage periods	49
L12	ADD	only about 7% of farms adopt the sewage anaerobic + aerobic standard discharge or recycling mode	Assume 50% adoption rate of additives during manure storage periods	
L13	ACP	Limited, commercial compost production is subsidised in several provinces. A few of the farms are equipped with anaerobic digestion, but most of them have been abandoned for high operating cost. 60% for Mixed system and 62% for industrial system	Assume 100 % implementation rate of farm-scale "anaerobic fermentation-aerobic compost" and "high-temperature aerobic compost" to recover biogas energy or produce high-efficiency organic fertilizer	28, 60
L14	BS	Limited, most smallholder farmers applying livestock manure by hand-spreading.	Assume 20% adoption rate of band application	49
L15	INC	0% for grazing system; 30% for mixed system and 50% for industrial system	Assume 30% adoption rate of manure incorporation as a whole	55, 60
L16	INJ	Limited. 5% for mixed system and 8% for industrial system	Assume 50% adoption rate of manure injection to reduce NH_3 emission	60
L17	IR	By 2018, the effective irrigated area of farmland in China was 6.8 million km^2.	Irrigation immediately following manure application. Water-saving irrigation will account for more than 60% of the effective irrigated area.	2
L18	AGT	Cows on pastures in winter is less than 40%	Increase cows on pastures in winter to 60%<br>Increasing the grazing period for cattle, sheep and goat	31

Supplementary Table 6 Effects of NH_3 control options on yield change, and emission of CH_4, N_2O and total GHG

NH_3 control options	Code	Yield change	CH_4 emission [a]	N_2O emission [b]	GHG emission change [c] (CO_2-eq)	Main reference
Urea substitution	C1	NA	0	NA	NA	Zhang et al. (2018)[61]<br>Xia et al. (2016)[4]<br>Pan et al. (2016)[3]<br>Wang et al., (2014)[37]<br>Huang and Tang (2010)[62]<br>Wang et al. (2020)[63]<br>Lu et al. (2000)[64]
Enhanced efficiency fertilizer (CRF)	C2	+	0	–	-38.3%	
Enhanced efficiency fertilizer (UI)	C2	+	0	–	-27.7%	
Optimizing N rate	C4	+	0	–	-31.2%	
Reducing basal fertilizer	C5	0	NA	NA	NA	
Increasing splitting frequency	C6	+	0	–	-5.41%	
Chemical fertilizer deep placement	C7	+	0	–	-14.6%	
Improved irrigation	C8, L17	NA	–	NA	NA	
Soil amendment	C9	+	0	NA	NA	
Diet manipulation	L1, L2, L3	0	–	–	-5%	Cao et al., (2019)[65]<br>Wang et al. (2018)[66]<br>Wang et al. (2017)[67]<br>Agyarko-Mintah et al. (2017)[68]<br>Hou et al. (2016)[20]<br>Hou et al. (2015)[28]
Housing adaptations	L4, L5, L7, L8	NA	–	–	-49%	
Air purification	L6	NA	0	+	NA	
Covered storage	L11	NA	+	+	+24%	
Manure acidification(additives)	L12	NA	–	–	-18%	
Manure composting	L13	NA	+	+	+13%	
Band application	C3, L14	NA	–	–	-3%	
Manure Incorporation	C3, L15	NA	+	+	+4%	
Manure Injection	C3, L16	NA	+	+	+16%	
Grazing	L18	0	–	–	-33%	

Note: '+', '-' and '0' indicate an increase, decrease and no change in emissions after application of control option, "NA" means not applicable or not available; [a] CH_4 emissions from rice cultivation, enteric fermentation and manure management; [b] N_2O emissions include both direct and indirect N_2O emissions from the application of synthetic fertilizers, organic manure and crop residues; [c] The overall GHG emissions are presented as kg CO2-eq, using the default values of 298 kg CO_2-eq for N_2O emissions and 34 kg CO_2-eq for CH_4 emissions (IPCC, 2013)[69].

Supplementary Table 7 Recommended combinations of mitigation options for crops and animals in China

	Available combinations of mitigative options	NH_3	GHG	Reference
**Planting type**				
Rice	RNF+ EENF+ organic fertilizer + biochar+ deep placement + integration of water and fertilizer	55-73%	6-45%	[38 41, 70-72]
Wheat	RNF +EENF+ manure incorporation+ split fertilization	51-83%	10-40%	[53, 73]
Maize	RNF + EENF + UI+ deep placement	39-81%	16-44%	[74]
Beans	RNF + organic fertilizer+ split + deep placement + irrigation	22-38%	NA	[11]
Tubers	RNF+ organic fertilizer + deep placement + integration of water and fertilizer	25-46%	NA	[11]
Cotton	RNF+ CRF+ organic fertilizer + integration of water and fertilizer	35-83%	21-36%	[75, 76]
Oil crops	RNF+ CRF+ organic fertilizer +deep placement+ integration of water and fertilizer	27-49%	NA	[11]
Sugar crops	RNF+ UI + residue management	45-70%	NA	[77, 78]
Fruits	RNF+ organic fertilizer+ incorporation	20-40%	16-46%	[2, 11]
Vegetable	RNF+ organic fertilizer + biochar+ incorporation	30-60%	10-45%	[7, 79-81]
**Animal type**				
Dairy cattle	LCP diet+ housing adaption + improve clean frequency + manure acidification+ incorporation	36-61%	2-15%	[82-84]
Beef cattle	LCP diet+ UI additive + manure scraper+ natural crust+ increase the grazing period	40-61%	5-11%	[27, 85]
Sheep and goat	Feed additives+ housing adaption+ grazing+ manure recycle to cropland	32-62%	3-10%	[86, 87]
Sow	LCP diet+ phase feeding + housing adaption +manure injection/incorporation	38-68%	9-47%	[14, 15]
Hog	LCP diet+ phase feeding + air-scrubbing systems + slurry and solid manure separation storage + injection/incorporation	38-68%	9-47%	[14, 15, 30]
Laying hen	LCP diet+ phase feeding + air-scrubbing systems+ ecological fermentation bed + slurry anaerobic storage + manure incorporation	61-85%	10-30%	[88, 89]
Other poultry	LCP diet + probiotics additives+ dry excrement +belts conveyors+ compost biofilter+ manure incorporation	60-89%	10-30%	[22, 89]
Rabbit	Feeding diets with yucca extract or probiotic +housing adaption+ manure treatment	42-57%	10-30%	[90, 91]
Horse/donkey/mule	Diet manipulation + peat bedding + compost+ grazing management	24-41%	NA	[92, 93]
Camel	Housing adaption+ grazing management	6-16%	NA	[94, 95]

Note: RNF refers to reduced use of urea-based N fertilizer; EENF refers to Enhanced Efficiency Nitrogen Fertilizer; SRF represents slow-released fertilizer, oil crops refer to peanut and rapeseed, sugar crops represent the sugar cane and sugar beets; LCP diet stands for low crude protein diet for indoor animals; all the above set of measures have been proposed as most promising measures for both mitigating NH_3 and GHG emissions from agricultural systems according to previous field experiment, meta-analyses and Chinese farming practices.

Supplementary Table 8 Population and social-economic development in China during 2020-2050

	2015	2020	2025	2030	2035	2040	2045	2050
Population (billion)	1.37	1.42	1.43	1.44	1.45	1.43	1.42	1.38
GDP (trillion US$)	11	17	23	32	42	49	52	55
PGDP (1000 US$）	8	12	16	22	29	34	37	40
Urbanization (%)	56	60	66	70	72	73	74	75

Data source: World bank[96]; FAO[97]; NBSC[98]

Supplementary Table 9 Food consumption under BAU and DIET scenario

Food consumption (kg/y/cap)	2015	2020	2025	2030	2035	2040	2045	2050
BAU-grain	127	120	115	110	105	100	95	90
BAU-fruits	85	100	105	110	115	120	125	130
BAU-vegetables	98	100	107	114	121	128	135	142
BAU-beans	8	8	8	8	8	8	8	8
BAU-livestock meat	35	40	45	50	55	60	65	70
BAU-fish	11	12	15	18	21	24	27	30
BAU-eggs	10	11	12	13	14	15	16	17
BAU-milk	12	15	25	35	45	55	65	75
DIET-grain	127	118	118	118	118	118	118	118
DIET-fruits	85	100	102	104	106	108	110	112
DIET-vegetables	98	100	105	110	115	120	125	130
DIET-beans	8	9	9	9	10	10	11	11

DIET-livestock meat	35	40	40	39	39	38	38	37
DIET-fish	11	11	12	13	14	15	16	17
DIET-eggs	10	11	12	12	13	13	14	14
DIET-milk	12	12	22	32	42	52	62	72

Note: the prediction of food consumption under BAU scenario is based on current high-income countries diet structure (animal food N ratio=60%); the prediction of food consumption under DIET scenario is based on the Dietary guidelines for Chinese residents[99] (animal food N ration=40%).

Supplementary Table 10 Projection of crop production (million t) under different scenarios

**Crops**	**2015**	**2020**	**2025**	**2030**	**2035**	**2040**	**2045**	**2050**
BAU-Rice	208	220	226	230	231	229	226	220
BAU-Wheat	13	138	140	140	137	133	128	122
BAU-Maize	225	270	299	328	354	377	399	418
BAU-Beans	16	19	19	19	19	19	19	18
BAU-Tubers	33	29	29	30	30	29	29	28
BAU-Cotton	60	35	36	36	36	36	35	34
BAU-Oil crops	35	5	5	5	5	5	5	5
BAU-Sugar crops	125	122	123	125	124	123	121	119
BAU-Vegetable	785	747	808	870	921	963	1000	1030
BAU-Fruits	274	268	279	295	308	318	326	332
DIET-Rice	208	214	220	226	229	230	230	229
DIET-Wheat	13	133	136	140	142	143	143	142
DIET-Maize	225	260	267	274	278	279	279	277
DIET-Beans	16	20	21	22	23	24	25	25
DIET-Tubers	33	29	29	30	30	29	29	28
DIET-Cotton	60	35	36	36	36	36	35	34
DIET-Oil crops	35	5	5	5	5	5	5	5
DIET-Sugar crops	125	122	123	125	124	123	121	119
DIET-Vegetable	785	747	793	839	875	903	926	943

DIET-Fruits	274	268	271	279	284	286	287	286

Note: REC and NUE scenarios are assumed to have the same crop production with BAU scenario, while ALL scenario has the same crop production with DIET scenario.

Supplementary Table 11 Projection of cropping area (million ha) under different scenarios

Item	2015	2020	2025	2030	2035	2040	2045	2050
BAU-Rice	30.2	31.0	30.9	30.6	29.7	28.6	27.4	25.9
BAU-Wheat	24.1	24.4	23.4	22.3	20.8	19.2	17.6	15.9
BAU-Maize	38.1	43.7	46.0	48.0	49.2	49.9	50.2	50.1
BAU-Beans	8.9	10.1	9.8	9.6	9.1	8.7	8.2	7.7
BAU-Tubers	8.8	7.6	7.5	7.4	7.2	6.9	6.6	6.3
BAU-Cotton	3.8	13.4	12.8	12.3	11.7	11.0	10.3	9.6
BAU-Oil crops	14.0	3.3	3.1	2.9	2.7	2.4	2.2	2.0
BAU-Sugar crops	1.7	1.7	1.7	1.7	1.7	1.6	1.6	1.5
BAU-Vegetable	22.0	19.9	20.5	21.0	21.1	21.0	20.8	20.4
BAU-Fruits	12.8	11.9	11.8	11.9	11.8	11.6	11.3	11.0
DIET-Rice	30.2	30.2	30.1	30.0	29.5	28.7	27.9	26.9
DIET-Wheat	24.1	23.4	22.9	22.4	21.6	20.6	19.6	18.5
DIET-Maize	38.1	41.9	41.0	40.1	38.6	36.9	35.1	33.2
DIET-Beans	8.9	10.8	11.0	11.1	11.1	11.0	10.8	10.5
DIET-Tubers	8.8	7.6	7.5	7.4	7.2	6.9	6.6	6.3
DIET-Cotton	3.8	13.4	12.8	12.3	11.7	11.0	10.3	9.6
DIET-Oil crops	14.0	3.3	3.1	2.9	2.7	2.4	2.2	2.0
DIET-Sugar crops	1.7	1.7	1.7	1.7	1.7	1.6	1.6	1.5
DIET-Vegetable	22.0	19.9	20.1	20.3	20.1	19.7	19.3	18.6
DIET-Fruits	12.8	11.9	11.5	11.3	10.9	10.4	10.0	9.4

Note: REC and NUE scenarios are assumed to have the same cropping areas with BAU scenario, while ALL scenario has the same cropping areas with DIET scenario.

Supplementary Table 12 Projection of livestock population (million head) under different scenarios

Livestock	2015	2020	2025	2030	2035	2040	2045	2050
BAU-Dairy cow	15.1	13	22	32	40	49	57	64
BAU-Beef cattle	46.7	51	73	93	110	124	135	144
BAU-Sheep and goat	267	296	300	303	302	299	294	288
BAU-Sow	50.0	52	56	58	61	62	63	64
BAU-Hog	663	739	794	835	865	887	904	915
BAU-Laying hen	1751	1986	2189	2396	2574	2727	2864	2978
BAU-Broiler	9110	10610	12528	14484	16264	17884	19380	20702
BAU-Laying duck	328	372	410	449	483	511	537	558
BAU-Duck	2278	2653	3132	3621	4066	4471	4845	5175
BAU-Laying goose	109	124	137	150	161	170	179	186
BAU-Goose	683	796	940	1086	1220	1341	1454	1553
BAU-Rabbit	524	340	340	340	340	340	340	340
BAU-Horse	5.91	3.5	3.5	3.5	3.5	3.5	3.5	3.5
BAU-Donkey	5.42	2.6	2.6	2.6	2.6	2.6	2.6	2.6
BAU-Mule	2.10	1.0	1.0	1.0	1.0	1.0	1.0	1.0
BAU-Camel	0.36	0.4	0.4	0.4	0.4	0.4	0.4	0.4
DIET-Dairy cow	15.1	11	20	29	38	46	54	62
DIET-Beef cattle	46.7	44	44	43	43	42	41	39
DIET-Sheep and goat	267	296	300	303	302	299	294	288
DIET-Sow	50.0	51	51	51	50	49	47	46
DIET-Hog	663	730	728	726	715	698	678	655
DIET-Laying hen	1751	1986	2098	2212	2298	2363	2416	2453
DIET-Broiler	9110	11169	11146	11120	10947	10685	10382	10026
DIET-Laying duck	328	372	393	415	431	443	453	460

DIET-Duck	2278	2792	2786	2780	2737	2671	2596	2506
DIET-Laying goose	109	124	131	138	144	148	151	153
DIET-Goose	683	838	836	834	821	801	779	752
DIET-Rabbit	524	340	340	340	340	340	340	340
DIET-Horse	5.91	3.5	3.5	3.5	3.5	3.5	3.5	3.5
DIET-Donkey	5.42	2.6	2.6	2.6	2.6	2.6	2.6	2.6
DIET-Mule	2.10	1.0	1.0	1.0	1.0	1.0	1.0	1.0
DIET-Camel	0.36	0.4	0.4	0.4	0.4	0.4	0.4	0.4

Note: Populations of horse, donkey, mule and camel are assumed to remain stable during 2020-2050. REC and NUE scenarios are assumed to have the same livestock population with BAU scenario, while ALL scenario has the same livestock population with DIET scenario.

Supplementary Table 13 Projection of total N fertilizer use and average application rate

	2015	2020	2025	2030	2035	2040	2045	2050
Total N fertilizer use (Tg)								
BAU	30.8	30.3	30.3	30.3	30.3	30.2	30.1	30.0
DIET	30.8	27.7	26.9	27.2	27.1	26.8	26.4	25.9
NUE	30.8	28.8	24.9	23.6	21.9	20.6	19.3	17.6
REC	30.8	27.7	25.6	23.9	21.2	17.8	14.4	12.2
ALL	30.8	20.9	17.4	14.6	11.6	8.6	6.7	5.2
N fertilizer rate (kg/ha) [a]								
BAU-Rice	173	150	150	150	150	150	150	150
BAU-Wheat	214	137	137	137	137	137	137	137
BAU-Maize	183	140	140	140	140	140	140	140
BAU-Beans	48	45	45	45	45	45	45	45
BAU-Tubers	206	200	200	200	200	200	200	200
BAU-Cotton	291	290	290	290	290	290	290	290
BAU-Oil crops	117	115	115	115	115	115	115	115
BAU-Sugar cane	342	342	342	342	342	342	342	342
BAU-Sugar beet	195	195	195	195	195	195	195	195
BAU-Vegetable	298	298	298	298	298	298	298	298
BAU-Fruits	445	400	400	400	400	400	400	400

[a] Under BAU scenario and “Zero increase” program, we assume future N fertilizer rates remain constant during 2020-2050.

Supplementary Table 14 Optimal application rates of chemical N fertilizer in China

	Rice	Wheat	Maize	Beans	Tubers	Cotton	Oil crops	Sugar cane	Sugar beet	Fruits (apple)	Vegetables
Optimal N application rate (kg N /ha) [a]	150	155	152	35	135	219	100	314	273	304	283
Actual N application rate (kg N /ha) [b]	173	213	183	45	160	296	124	416	324	363	303
Urea	59%	57%	59%	30%	60%	64%	47%	77%	58%	44%	58%
ABC	7%	3%	4%	5%	5%	9%	9%	7%	10%	1%	12%
other kinds of N fertilizer	0%	0%	0%	0%	1%	1%	0%	0%	0%	1%	0%
NPK	33%	39%	37%	65%	34%	26%	45%	16%	32%	54%	30%
Farmyard manure application rate (kg/ha)	61	113	1048	19	750	85	58	34	116	984	1252
proportion of fields applying organic fertilizer	11.9%	34.5%	20.5%	20%	35%	20%	20%	30%	30%	50%	56%

[a] the optimal N application rates are weighted by regional recommended values, which are derived from Guidelines for major crop fertilization in China[11].

[b] the actual N fertilizer application rates and source contribution (%) of different crops were collected from the China Agricultural Products Cost-Benefit Yearbooks (NDRC, 2000-2018).

Supplementary Table 15 Cost and labour involvement of crop farming in 2015

Items (Unit: CNY)	rice	wheat	maize	bean	cotton	sugar cane	sugar beet	vegetables	fruits	oil crops
**A. Direct material and service cost per ha**	479	420	5643	202	620	778	609	1403	1768	335
(1) Direct expenses	458	410	5474	189	570	518	504	1124	1474	327
1.Seed cost	55	66	852	37	55	0	90	157	0	93
2.Fertilizer cost	122	143	1968	46	203	343	154	284	493	105
3.Organic manure cost	9	13	168	3	11	6	9	178	250	10
4.Pesticide cost	51	20	249	16	71	59	14	117	261	24
5.Agri. film cost	4	0	70	0	31	4	16	149	24	4
6.Renting and operation cost	205	163	2094	84	174	89	190	145	144	84
Machine renting and operation	176	131	1680	80	99	57	122	88	59	67
Irrigation and drainage	21	30	323	3	73	2	68	51	85	7
Water cost	10	6	85	0	25	2	30	15	12	1
7.Fuel and power cost	3	1	6	0	4	3	1	12	29	1
8.Technical service cost	0	0	0	0	1	0	0	1	2	0
9.Tool and material cost	6	3	49	2	17	11	29	69	231	4
10.Maintenance and repair cost	2	1	18	1	3	3	1	12	39	1
11.Other direct cost	0	0	0	0	0	0	0	1	0	0
(2) Indirect expenses	20	10	169	13	50	260	105	278	293	7
1. Fixed assets depreciation cost	8	3	56	1	20	140	3	171	90	5
2. Insurance cost	9	5	90	7	19	7	2	4	14	2
3. Management cost	3	0	15	4	0	0	3	6	76	0
4. Financial cost	0	0	0	0	6	0	0	1	0	0
5. Sales fee	1	1	7	1	6	113	97	96	113	1
**B. Labour cost per ha**	509	364	7031	215	1388	1169	710	2597	3254	631
1.Family labour cost	448	352	6708	196	1182	660	534	1879	1939	619
Labour input days	6	5	86	3	15	8	7	24	25	8

Labour daily wage	78	78	1170	78	78	78	78	78	78	78
2.Hiring labour cost	61	12	323	19	206	509	176	717	1314	12
Labour input days	0	0	3	0	2	6	2	8	13	0
Labour daily wage	126	93	1503	116	108	90	116	86	105	89

Note：CNY refers to Chinese Yuan; Data source: (NCDR,2018)[33]

Supplementary Table 16 Cost and labour involvement of livestock farming in 2015

Item (Unit: CNY)	Unit	Pig	Dairy cattle	Beef cattle	Sheep and goat	Broiler (per 100)	Layer (per 100)
**A. Material and service cost per animal**	CNY	1376	15045	7499	571	2284	13875
(1) Direct expenses	CNY	1361	12626	5705	343	279	13672
1. Cub cost	CNY	425	-	1286	140	1833	2811
2. Refined feed cost	CNY	872	8493	423	62	3	10510
3. Silage crude feed cost	CNY	16	3313	14	4	1	0
4. Feed processing cost	CNY	5	42	4	2	4	11
5. Water cost	CNY	2	47	9	2	36	14
6. Fuel and power costs	CNY	6	180	9	2	12	71
Electricity	CNY	4	121	1	0	24	61
Coal cost	CNY	1	43	0	0	0	10
Other fuel costs	CNY	1	15	21	6	75	0
7. Medical and epidemic prevention cost	CNY	17	191	22	7	28	138
8. Death loss cost	CNY	12	119	0	0	5	88
9. Technical service cost	CNY	0	14	7	2	9	2
10. Tools and materials cost	CNY	3	66	6	2	9	11
11. Repair and maintenance costs	CNY	2	49	0	0	1	16
12. Other direct costs	CNY	1	114	40	9	35	0
(2) Indirect expenses	CNY	15	2419	30	6	26	203
1. Fixed assets depreciation cost	CNY	10	2115	0	0	0	125
2. Insurance cost	CNY	1	44	0	0	2	2
3. Management cost	CNY	1	127	0	0	0	21
4. Financial cost	CNY	0	38	10	3	6	5
5. Sales fee	CNY	2	96	1011	422	265	50
**B. Labour cost per animal**	CNY	343	3348	934	416	214	1234
1.Family labour cost	CNY	318	2256	12	5	3	877

Labour input days	day	4	29	78	78	78	11
Labour daily wage	CNY	78	78	76	6	50	78
2.Hiring labour cost	CNY	25	1092	1	0	1	357
Labour input days	day	0	11	104	105	100	4
Labour daily wage	CNY	89	100	0	0	0	93

Note：CNY refers to Chinese Yuan; Data source: (NCDR,2018) [100]

Supplementary Table 17 Assumed the growth rate of farming cost per five-year (%) for crops during 2020-2050

	**rice**	**wheat**	**maize**	**bean**	**cotton**	**sugar cane**	**sugar boot**	**vegetable**	**fruits**	**oil crops**
**A. Direct material and service cost per ha**										
**(1) Direct expenses**	**5%**	**5%**	**4%**	**-1%**	**12%**	**5%**	**17%**	**8%**	**-12%**	**4%**
1.Seed cost	4%	5%	4%	-1%	8%	-9%	15%	13%	-8%	4%
2.Fertilizer cost	13%	13%	3%	4%	2%	-20%	12%	17%	0%	0%
3.Organic manure cost	-4%	-4%	-5%	-11%	-1%	0%	4%	2%	-1%	-1%
4.Pesticide cost	3%	35%	7%	41%	3%	-30%	46%	52%	-17%	4%
5.Agri. film cost	4%	20%	11%	7%	4%	18%	0%	6%	-7%	11%
6.Renting and operation cost	1%	0%	2%	0%	15%	-1%	40%	1%	-17%	-2%
Machine renting and operation	8%	8%	15%	2%	17%	-1%	25%	6%	0%	14%
Irrigation and drainage	13%	11%	19%	2%	15%	9%	8%	7%	-3%	24%
Water cost	-4%	-1%	10%	3%	26%	-32%	70%	7%	3%	-7%
7.Fuel and power cost	-29%	-21%	-20%	-18%	-34%	-14%	0%	-11%	-50%	-16%
8.Technical service cost	51%	43%	41%	-6%	54%	0%	-4%	12%	-26%	68%
9.Tool and material cost	75%	0%	0%	-50%	-14%	0%	0%	89%	-41%	0%
10.Maintenance and repair cost	3%	4%	-2%	-2%	67%	3%	43%	-2%	-2%	6%
11.Other direct cost	-1%	-8%	-7%	-1%	62%	7%	20%	2%	-43%	12%
**(2) Indirect expenses**	**50%**	**0%**	**0%**	**0%**	**0%**	**-30%**	**20%**	**4%**	**-50%**	**0%**
1. Fixed assets depreciation cost	19%	14%	22%	-2%	13%	12%	28%	-8%	-27%	12%
2. Insurance cost	9%	-2%	6%	35%	56%	57%	50%	1%	9%	10%
3. Management cost	31%	39%	51%	14%	45%	57%	21%	-8%	-10%	-50%
4. Financial cost	20%	-31%	-9%	-26%	0%	-40%	61%	21%	-41%	0%
5. Sales fee	-38%	0%	-38%	-50%	28%	0%	13%	56%	-36%	0%
**B. Labour cost per ha**										
1.Family labour cost	7%	12%	5%	11%	8%	9%	31%	14%	12%	14%

Labour input days	6%	11%	6%	12%	2%	7%	42%	-4%	22%	14%
Labour daily wage	-12%	-9%	-12%	-2%	-15%	-11%	12%	-19%	-1%	-7%
2.Hiring labour cost	24%	24%	24%	24%	24%	24%	24%	24%	24%	24%
Labour input days	13%	46%	-8%	4%	65%	11%	8%	79%	-3%	12%
Labour daily wage	-3%	44%	-16%	-10	30%	-3%	-1%	34%	-15%	-6%

Note: projections are based on the historical data derived from China Agricultural Products Cost-Benefit Yearbook (2000-2018)[100]

Supplementary Table 18 Assumed growth rate of farming cost per five-year (%) for animals during 2020-2050

Items	Unit	Pig	Dairy cattle	Beef cattle	Sheep and goat	broiler	Layer
**A. Material and service cost per animal**	CNY	4%	2%	-1%	-5%	-3%	-6%
(1) Direct expenses	CNY	4%	1%	-1%	-6%	-3%	-7%
1. Cub cost	CNY	20%	0%	0%	-6%	2%	-2%
2. Refined feed cost	CNY	-3%	-2%	-4%	-7%	-5%	-9%
3. Silage crude feed cost	CNY	-12%	9%	-8%	2%	0%	0%
4. Feed processing cost	CNY	-7%	-3%	4%	1%	-9%	-19%
5. Water cost	CNY	5%	9%	7%	-9%	-5%	-8%
6. Fuel and power costs	CNY	-2%	6%	-3%	-4%	2%	9%
Electricity	CNY	5%	11%	0%	1%	15%	13%
Coal cost	CNY	-20%	-11%	-19%	-23%	-3%	-9%
Other fuel costs	CNY	3%	22%	0%	0%	0%	0%
7. Medical and epidemic prevention cost	CNY	2%	8%	-6%	5%	-1%	5%
8. Death loss cost	CNY	-2%	-10%	-1%	3%	-1%	2%
9. Technical service cost	CNY	-19%	-6%	-20%	-20%	-4%	20%
10. Tools and materials cost	CNY	-2%	12%	-2%	-3%	9%	10%
11. Repair and maintenance costs	CNY	-1%	0%	-13%	-8%	7%	5%
12. Other direct costs	CNY	-1%	-1%	0%	0%	0%	-50%
(2) Indirect expenses	CNY	7%	8%	2%	2%	6%	13%
1. Fixed assets depreciation cost	CNY	2%	10%	6%	2%	9%	11%
2. Insurance cost	CNY	75%	30%	0%	0%	-50%	50%
3. Management cost	CNY	7%	-7%	-9%	10%	2%	25%
4. Financial cost	CNY	-14%	-9%	0%	0%	-11%	-4%
5. Sales fee	CNY	4%	2%	-6%	2%	-5%	11%
**B. Labour cost per animal**	CNY	3%	9%	7%	8%	9%	0%
1.Family labour cost	CNY	3%	10%	10%	11%	12%	14%

Labour input days	day	-7%	-1%	-1%	0%	1%	2%
Labour daily wage	CNY	11%	11%	11%	11%	11%	11%
2.Hiring labour cost	CNY	3%	8%	-18%	-27%	-1%	-3%
Labour input days	day	-8%	-5%	-25%	-31%	-11%	-13%
Labour daily wage	CNY	12%	14%	14%	11%	13%	14%

Note：projections of the growth rates are based on historical data derived from China Agricultural Products Cost-Benefit Yearbook (2000-2018)[33].

Supplementary Table 19 Comparison of farm size, labour cost among China and other countries

Region	The percentage of different farm size (2015)							Labour cost (2015) (US$ per hour)
	0-1 ha	1-2ha	2-5ha	5-10ha	10-20ha	20-50ha	>50ha	
Denmark	0%	2%	2%	16%	20%	30%	31%	36.4
Netherland	0%	16%	15%	16%	17%	28%	8%	20.2
Germany	0%	8%	17%	16%	19%	24%	17%	9.74
EU_{27}	0%	10%	16%	17%	15%	20%	22%	17.0
USA	0%	0%	11%	10%	14%	22%	44%	13.1
Canada	0%	2%	3%	4%	5%	14%	72%	13.3
China	93%	5%	2%	-	-	-	-	2.5

data source: (FAO,2019), EEA[101], NBSC[98]

Supplementary Table 20 Parameterization scheme of WRF-CMAQ

**Parameterization scheme**	**CMAQ**
Version	5.0.2
Grid nesting	Single-layer grid
Horizontal resolution	20 km
Vertical layers	14
Gas phase chemistry	CB05
Aerosol chemistry	AERO5
Photochemical rate	In-line
The wind dust	Off
The boundary conditions	Default
The initial conditions	Restart every single day
**Parameterization scheme**	**WRF**
Version	3.4
Microphysical schemes	WSM6
Longwave Radiation	New Goddard scheme
Shortwave Radiation	RRTM
Surface layer	Pleim Xiu
Interaction with Earth's surface	Pleim Xiu
Boundary layer	ACM2
Cumulus convection	Kain-Fritsch

Supplementary Note 1. Selection of NH_3 mitigation measures

Agricultural NH_3 emissions in this study refer to the NH_3 emissions from crop and livestock production systems. The mitigation potentials of livestock production and crop farming are described separately after taking into account their interaction through manure recycling to fields. Given the inherent differences among the various crops and livestock types, here we assess NH_3 mitigation potential by crop and animal types to explore the feasible national emission reduction target[2]. For example, options for reducing NH_3 losses from poultry housing and manure storage focus on rapidly drying and transferring the manure to the storage area. Measures for slurry storage from pigs and cattle generally aim at minimizing contact between manure surface and air. According to the agricultural characteristics and farming practice in China, 10 main crops and 10 animal types in China were selected for sector specific NH_3 mitigation assessment.

In fact, not all measures can be applied to 100%, some techniques are restricted in applicability by their effectiveness or by practical limitation[14]. These limitations may be of very different natures, including local climate, soil conditions (pH, slope), farm size, financial and technical limitations[14]. Therefore, implementation of NH_3 abatement measures should follow their applicability and be adjusted to local conditions[2]. Supplementary Tables 3 and 4 summarize the practical consideration of NH_3 mitigation options applied to cropland and animal production in China. National policy and plans have been taken into consideration to determine the parameters or implementation level of different options in the future[33, 42, 47, 55, 102-104]. Supplementary Table 5 lists the current farming practice in China and the applicability of the selected measures.

Note that some individual options (e.g. manure covered storage) do not really remove NH_3 but merely preserve N in the manure, which may be emitted at later stages, (e.g. the stage of application[105]). Therefore, these measures should be used in conjunction with other options to enhance the mitigation efficiency by positive measure interactions, e.g. manure treatment should generally be coupled with improved manure application methods to avoid N loss during application. Supplementary Table 6 summarizes the possible effects of NH_3 mitigation measures on GHG (N_2O and CH_4) emissions. Some mitigation options for NH_3 reduction may induce GHG emissions at certain stage. For example, the application of manure and straw cover, or injection of liquid fertilizers to reduce NH_3 volatilization may lead to increased emissions of methane (CH_4) by the anaerobic decomposition of manure, and of nitrous oxide (N_2O) through nitrification and denitrification in livestock manure and urine[30]. However, optimal combinations of different measures could offset the stage-specific side-effects and achieve both NH_3 mitigation and total GHG mitigation[28, 66, 67]. The selected packages of measures in Supplementary Table 7 could improve, or at least maintain, crop yields or animal productivity according to the results of existing studies and meta-analyses. However, due to limited Chinese-specific experimental data of yield change, the economic benefits of crop and animal productivity improvement are not quantified in the cost-benefit analysis.

Supplementary Note 2. Reduction efficiency of mitigation measures for crop farming

The abatement effect of single option is easy to access due to a lot of previous field

experiments, measurements and meta-analyses[2]. However, in practice, implementation of a single option alone has limitations to its abatement effectiveness and cost. For example, the use of NBPT (a kind of Urease inhibitor) alone may not be sufficiently effective in inhibiting NH_3 emissions, while NBPT with recommended N fertilizer type and optimal irrigation management may achieve the desired results of decreasing N losses and increasing N use efficiency[106]. Note that the abatement rate and cost-effectiveness of individual measures may change when applied with other measures jointly, so the actual abatement efficiency and cost-effectiveness of the combined packages of mitigation options need to be identified or recalibrated to explore the maximum mitigation potential, and to inform the best cost-effective abatement strategies[2].

Due to limited information on the effectiveness of combinations of measures for NH_3 mitigation in cropping systems, here the interactions are addressed by assigning implementation priorities to selected mitigation options. For example, if measure C1 and C4 allow N application rates to decrease from 200 kg ha^{-1} to 100 kg ha^{-1}, the mitigation effect of deep placement (measure C7) will be based on the N rate of 200 kg ha^{-1}. The potential of adding organic manure (measure C3) to rice paddies is quantified under the improved irrigation by measure C8. If there are no supporting data available from previous field experiments or meta-analysis results for quantitative analysis of the NH_3 mitigation potential in cropping system, the combined efficiency for a package of two (A+B) and three (A+B+C) mitigation options was assessed following the below Equation (1-2):

$$\eta_{A+B} = \eta_A + (1 - \eta_A)\eta_B \quad (1)$$

$$\eta_{A+B+C} = \eta_A + (1 - \eta_A)\eta_B + [1 - (\eta_A + (1 - \eta_A)\eta_B)]\eta_c \quad (2)$$

where A, B, C are the control technologies included in the combination, $\eta_{A,B,C}$ is the reduction efficiency of a given mitigation option.

Supplementary Note 3. Reduction efficiency of mitigation measures for livestock production

NH_3 emissions from livestock production are based on the concept of the N mass flow balance in livestock system (Supplementary Figure 2). NH_3 emission at each stage of the management systems, including housing, storage, grazing and application to the land was calculated following Equation (3-6). It is therefore clear that the volume of NH_3 emissions from the later stages are affected by emissions from previous stages. For example, reducing NH_3 volatilization at previous stage, such as covered storage stage, will likely create a more N-rich waste and therefore greater potential of NH_3 loss at a later stage, such as land application[107]. It makes sense to adopt low emission techniques during manure application. Thus, optimal package of mitigation options that covers all stages N flow need to be designed to systematically reduce NH_3 emissions.

In this study, results of meta-analyses or currently available research about combinations of multiple options in China are mainly referred to explore the maximum mitigation potential. For instance, combined NH_3 mitigation options on feed, housing,

manure storage, and land application could reduce the farm-scale NH_3 emission by up to 89.3% in broiler production systems[89]. Combined effect of the selected measure at a given stage on NH_3 emission factor was estimated following the Equation (7-10) when data is not available.

The livestock NH_3 emission factors:

$$ef_1 = \mathrm{Nx}_1 v_1 \quad (3)$$
$$ef_2 = \mathrm{N}x_1(1 - v_1)v_2 \quad (4)$$
$$ef_3 = \mathrm{N}x_1(1 - v_1 - (1 - v_1)v_2)v_3 \quad (5)$$
$$ef_4 = \mathrm{Nx}_4 v_4 \quad (6)$$

where $ef_{1,2,3,4}$ is the NH_3-N loss at different emission stages, i.e., housing (1), storage (2), application (3), and grazing (4), $\mathrm{N}x_{1,4}$ is the N excretion during housing (1) and grazing (4), $v_{1,2,3,4}$ is the N volatilization rates at specific emission stages.

$$ef_1' = \mathrm{Nx}_1 v_1 \eta_1 \quad (7)$$
$$ef_2' = \mathrm{N}x_1(1 - v_1\eta_1)v_2\eta_2 \quad (8)$$
$$ef_3' = \mathrm{N}x_1(1 - v_1\eta_1 - (1 - v_1\eta_1)v_2\eta_2)v_3\eta_3 \quad (9)$$
$$ef_4' = \mathrm{Nx}_4 v_4 \eta_4 \quad (10)$$

where $ef'_{1,2,3,4}$ is the new NH_3-N emission factors at specific emission stages, $\eta_{1,2,3,4}$ is the NH_3 reduction efficiency at specific emission stages.

For livestock production combinations of measures within a stage may interact, including overlapping application of measures with similar effects or subordinating relationships. For example, during animal housing, optimal bedding, floor adaptation and air purification could be applied separately or jointly. Information about the combined abatement efficiency within same stage is mainly collected from current research (i.e. field experiment, model simulation and meta-analysis)[20, 30].

Supplementary Note 4. Data source and projection of future agricultural activity under different mitigation pathways

The years 2000-2015 are used as the reference years in this study and 2020-2050 in five-year intervals are set as the target years. According to the definition, classification and applicability of previously selected mitigation options, five emission scenarios were proposed in this study and combined with CHANS and GAINS models to explore the mitigation potential and the costs and benefits of each mitigation pathway in the next 30 years (2020-2050).

For data sources, historical data for 2000-2015 such as population, urbanization, gross domestic product (GDP), land use, fertilizer use, crop/livestock production, and resource consumption in the agricultural sector of China, were mainly collected from the National Bureau of Statistics of China (NBSC)[100] and Food and Agriculture Organization of the United Nations (FAO) statistics[108]. Prediction of future agricultural activity data for 2020-

2050 is based on the N demand-supply balance framework (Supplementary Figure 3) i.e. N supply from crop and livestock production should meet the demands of human consumption. This framework combines various input drivers and parameters with previous scenario studies (e.g. Gu et al. (2015); Ma et al.(2019)[109]). National plans and regulations (e.g. Zero Increase Action Plan on Fertilizer Use by 2020[110]) are also considered in the prediction.

We first predicted the future human population, GDP, diet preference and urbanization; then, the demands of crop and animal feed were estimated; third, the required crop and livestock production, cropping area, fertilizer use and manure production were calculated for subsequent scenario analysis.

Most future prediction of basic activity data were directly derived from results of a series of models and scenarios derived from previous researches [109, 111, 112] where various sources were used, including national plans or targets (National population development plan (2016-2030)[113], National plan for agricultural modernization (2016-2020)[114]; National plan for sustainable agricultural development(2015-2030)[55]), which are the most reliable data sources in China. Meanwhile, associated data from international organizations including the FAO[108, 115], the World Bank[96], the IIASA[116] were also collected to support the projection of future agricultural activity.

To make the scenarios clearer we extracted the important activity level indicators, coefficients and parameters shown in Supplementary Tables 8 - 19. Under the BAU scenario we assume a population of 1.38 billion with an urbanization level of 75% and a PGDP of US$ 39,900 in 2050. The projection of the Chinese population in the future takes into account the universal two-child policy adopted in 2015[112, 117]. The predictions of GDP and PGDP in China are based on the past stable economic growth in China[96, 109, 111] and socio-economic models and data in IPCC scenarios[118]. Human food consumption would increase due to the population and PGDP growth[51, 119]. The projections of sowing area and crop productivity in China during 2020–2050 are based on requirements of the plant-based food and animal-based food consumption[120]. The forecast of fertilizer use is based on the fertilizer policy issued in 2015 “Zero-growth Action Plan” for chemical fertilizer application by 2020[55]. The NUEs and nutrient recycling rates of agricultural subsystems (e.g., cropland and livestock) are assumed to remain current level under BAU scenario. International trade (e.g. grain imports) are assumed to remain constant during 2020-2050.

It should be noted that there are numerous uncertainties in this scenario-based analysis of the future NH_3 emissions and mitigation in China. Although our estimates in this study are based on current best available information, the accuracy and robustness of our estimates are still limited by the quality of the data, the applicability of the mitigation options and the validity of the assumptions made in terms of future activity levels. Estimates of the combined effects of a group of mitigation options could also be an important source of uncertainty because the practical NH_3 removal efficiencies for NH_3 and implementation levels in the future are unattainable and the lack of validation and optimization will also bring large uncertainty in the predictions. In addition, considerable uncertainties are also recognized regarding the estimation of abatement costs and societal benefits of NH_3 mitigation. Such uncertainties are inevitable and need to be addressed and assessed with more bottom-up research in support of policies on agricultural NH_3 emissions.

Supplementary Note 5. WRF/CMAQ model simulation of $PM_{2.5}$ concentration to NH_3 emission control

A detailed sensitivity simulation of $PM_{2.5}$ reduction by NH_3 mitigation using the WRF-CMAQ model in China was conducted by Xu et al (2017)[1]. The main description and validation of WRF-CMAQ simulation are listed as follows:

(1) Simulation period: January, April, July and October of 2015, and the time interval of the output is 1h.

(2) Simulation area: the CMAQ model adopts the Lambert projection coordinate system, the central longitude is 103°E, the central latitude is 37°N, the two parallel latitudes are 25°N and 40°N, respectively. The horizontal simulation range for the X direction is -2690 to 2690 km, and for the Y direction it is -2150 to 2150 km with a grid spacing of 20 km. The whole of China was divided into 270×216 grids. A total of 14 pressure layers were set in the vertical direction, and the layer spacing gradually increased from bottom to top.

(3) Meteorological simulation: the meteorological field required by the CMAQ model is provided by the mesoscale meteorological model WRF. The WRF model and the CMAQ model adopt the same simulation period and spatial projection coordinate system, but the simulation range of WRF is larger than that of CMAQ. The horizontal simulation range for the X direction was -3600 km to 3600 km, and for the Y direction it was -2520 km to 2520 km, with a grid spacing of 20 km.

(4) Parameterization scheme of WRF-CMAQ was summarized in Supplementary Table 20.

The performance of WRF-CMAQ model simulation in simulating $PM_{2.5}$ chemical components at different geographic locations and times have been statistically evaluated in Xu et al. (2017), which generally shows good correlation of simulated ambient $PM_{2.5}$ chemical composition relative to observed values. The correlation coefficient between observed and simulated annual average $PM_{2.5}$ concentration was 0.82 ($n=302$, $p<0.05$), with normalized mean biases (NMB) of -21.67 and Normalized mean error (NME) of 29.49.

Responses of different compositions of $PM_{2.5}$ to NH_3 mitigation was investigated by WRF-CMAQ model simulation. Results showed that reducing NH_3 emissions could significantly decrease annual average concentration of nitrate, ammonium and $PM_{2.5}$ concentrations (Supplementary Figure 4). Sulfate concentration is not sensitive to NH_3 mitigation because of its low vapor pressure and thermodynamic stable nature[121, 122]. In contrast, nitrate concentration is very sensitive to changes in NH_3 emissions because its high saturated vapor pressure and thermal stability[123]. The availability of atmospheric NH_3 is one of the key factors determining the transformation of HNO_3 into NH_4NO_3 in China. This model result is also consistent with Liu et al. (2019)[122] and Xu et al. (2019)[123] simulated by WRF-Chem model.

References:

1. Xu, Y., *et al*. Sensitivity analysis of $PM_{2.5}$ pollution to ammonia emission control in China. China Environ. Sci. **37**, 2482-2491 (2017).

2. Ti, C., Xia, L., Chang, S.X. & Yan, X. Potential for mitigating global agricultural ammonia emission: A meta-analysis. Environ. Pollut. **245**, 141-148 (2019).

3. Pan, B., Lam, S.K., Mosier, A., Luo, Y. & Chen, D. Ammonia volatilization from synthetic fertilizers and its mitigation strategies: A global synthesis. Agric., Eco. & Environ. **232**, 283-289 (2016).

4. Xia, L., *et al*. Can knowledge-based N management produce more staple grain with lower greenhouse gas emission and reactive nitrogen pollution? A meta-analysis. Glob. Change. Biol. **23**, 1917-1925 (2017).

5. Ni, B., Liu, M., Lu¨, S., Xie, L. & Wang, Y. Environmentally Friendly Slow-Release Nitrogen Fertilizer. J. Agric. Food Chem. **59**, 10169-10175 (2011).

6. Xia, L., Lam, S.K., Yan, X. & Chen, D. How Does Recycling of Livestock Manure in Agroecosystems Affect Crop Productivity, Reactive Nitrogen Losses, and Soil Carbon Balance? Environ. Sci. Technol. **51**, 7450-7457 (2017).

7. Zhang, J., *et al*. Substituting organic manure for compound fertilizer increases yield and decreases NH_3 and N_2O emissions in an intensive vegetable production systems. Sci. Total. Environ. **670**, 1184-1189 (2019).

8. Goos, R.J. Effects of Fertilizer Additives on Ammonia Loss after Surface Application of Urea-Ammonium Nitrate Fertilizer. Commun. Soil. Sci. Plant Anal. **44**, 1909-1917 (2013).

9. Huang, S., *et al*. Effects of fertilizer management practices on yield-scaled ammonia emissions from croplands in China: A meta-analysis. Field Crops Res. **192**, 118-125 (2016).

10. Ju, X.T., *et al*. Reducing environmental risk by improving N management in intensive Chinese agricultural systems. Proc. Natl Acad. Sci. USA **106**, 3041-3046 (2009).

11. Zhang, F., Chen, X. & Chen, Q. *Guidelines for major crop fertilization in China* (China Agriculture Press, 2009).

12. Bittman, S., Dedina, M., Howard, C.M., Oenema, O. & Sutton, M.A.E. *Options for Ammonia Mitigation: Guidance from the UNECE Task Force on Reactive Nitrogen* (NERC/Centre for Ecology & Hydrology, Edinburgh, UK, 2014).

13. Chen, D., *et al*. A new cost-effective method to mitigate ammonia loss from intensive cattle feedlots: application of lignite. Sci. Rep. **5**, 16689 (2015).

14. Reis, S., Howard, C. & Sutton, M.A. *Costs of Ammonia Abatement and the Climate Co-Benefits* (Springer, 2015).

15. Sajeev, E.P.M., Amon, B., Ammon, C., Zollitsch, W. & Winiwarter, W. Evaluating the potential of dietary crude protein manipulation in reducing ammonia emissions from cattle and pig manure: A meta-analysis. Nutr. Cycl. Agroecosyst. **110**, 161-175 (2018).

16. Bittman, S., Dedina, M., Howard, C.M., Oenema, O. & Sutton, M.A. *Options for Ammonia Mitigation-Guidance from the UNECE Task Force on Reactive Nitrogen* (Centre for Ecology and Hydrology, Edinburgh, UK, 2014).

17. Loyon, L., *et al*. Best available technology for European livestock farms: Availability, effectiveness and uptake. J. Environ. Manag. **166**, 1-11 (2016).

18. Pierer, M., Amon, B. & Winiwarter, W. Adapting feeding methods for less nitrogen pollution from pig and dairy cattle farming: abatement costs and uncertainties. Nutr. Cycl. Agroecosyst. **104**, 201-220 (2016).

19. Shah, G.A., *et al*. Bedding additives reduce ammonia emission and improve crop N uptake after soil application of solid cattle manure. J. Environ. Manag. **209**, 195-204 (2018).

20. Hou, Y., Velthof, G.L., Lesschen, J.P., Staritsky, I.G. & Oenema, O. Nutrient Recovery and Emissions of Ammonia, Nitrous Oxide, and Methane from Animal Manure in Europe: Effects of Manure Treatment Technologies. Environ. Sci. Technol. **51**, 375-383 (2016).

21. Van der Heyden, C., Demeyer, P. & Volcke, E.I.P. Mitigating emissions from pig and poultry housing facilities through air scrubbers and biofilters: State-of-the-art and perspectives. Biosyst. Eng. **134**, 74-93 (2015).

22. Cao, Y., *et al*. Review on Ammonia Emission Mitigation Techniques of Crop-Livestock Production System. Zhongguo Nong Ye Ke Xue **51**, 566-580 (2018).

23. Liu, Z., Powers, W. & Mukhtar, S. A review of practices and technologies for odor control in swine production facilities. **30**, 477-492 (2014).

24. Baldé, H., *et al*. Ammonia emissions from liquid manure storages are affected by anaerobic digestion and solid-liquid separation. Agric. For. Meteorol. **258**, 80-88 (2018).

25. Chen, W., *et al*. Effects of different types of biochar on methane and ammonia mitigation during layer manure composting. Waste Manag. **61**, 506-515 (2017).

26. Cao, Y., *et al*. Acidification of manure reduces gaseous emissions and nutrient losses from subsequent composting process. J. Environ. Manage. **264**, 110454 (2020).

27. Wang, X., *et al*. Composting with negative pressure aeration for the mitigation of ammonia emissions and global warming potential. J. Clean. Prod. **195**, 448-457 (2018).

28. Hou, Y., Velthof, G.L. & Oenema, O. Mitigation of ammonia, nitrous oxide and methane emissions from manure management chains: a meta-analysis and integrated assessment. Glob. Change. Biol. **21**, 1293-1312 (2015).

29. Klimont, Z. & Winiwarter, W. Estimating costs and potential for reduction of Ammonia emissions from agriculture in the GAINS model. in *Costs of ammonia abatement and the climate co-benefits* 233-261 (Springer, 2015).

30. Hou, Y., Velthof, G.L. & Oenema, O. Mitigation of ammonia, nitrous oxide and methane emissions from manure management chains: a meta-analysis and integrated assessment. Glob. Change Biol. **21**, 1293-1312 (2015).

31. Bittman, S., Sheppard, S.C. & Hunt, D. Potential for mitigating atmospheric ammonia in Canada. Soil Use Manage. **33**, 263-275 (2017).

32. MOA. Scientific Application of Fertilizer to the Chief Crop 2019. http://www.moa.gov.cn/xw/zxfb/201903/t20190301_6173059.htm (2019).

33. NDRC. China Agricultural Products Cost-Benefit Yearbook (2000-2018). (2019).

34. Alibaba Groups. 1688 Wholesale website. https://www.1688wholesale.com/en/index.html (2019).

35. Mariano, E., de Sant Ana Filho, C.R., Bortoletto-Santos, R., Bendassolli, J.A. & Trivelin, P.C.O. Ammonia losses following surface application of enhanced-efficiency nitrogen fertilizers and urea. Atmos. Environ. **203**, 242-251 (2019).

36. Zhang, M., *et al*. Integration of urea deep placement and organic addition for improving yield and soil

properties and decreasing N loss in paddy field. Agric., Eco. & Environ. **247**, 236-245 (2017).

37. Wang, W., *et al*. Greenhouse gas mitigation in Chinese agriculture: Distinguishing technical and economic potentials. Glob. Environ. Change. **26**, 53-62 (2014).

38. He, T., *et al*. A two years study on the combined effects of biochar and inhibitors on ammonia volatilization in an intensively managed rice field. Agric. Ecosyst. Environ **264**, 44-53 (2018).

39. Keshavarz Afshar, R., Lin, R., Mohammed, Y.A. & Chen, C. Agronomic effects of urease and nitrification inhibitors on ammonia volatilization and nitrogen utilization in a dryland farming system: Field and laboratory investigation. J. Clean. Prod. **172**, 4130-4139 (2018).

40. Rochette, P., *et al*. Ammonia Volatilization and Nitrogen Retention: How Deep to Incorporate Urea? J. Environ. Qual. **42**, 1635-1642 (2013).

41. Li, J., *et al*. Combination of modified nitrogen fertilizers and water saving irrigation can reduce greenhouse gas emissions and increase rice yield. Geoderma **315**, 1-10 (2018).

42. The State Council. Action Plan for Prevention and Control of Water Pollution. http://www.gov.cn/zhengce/content/2015-04/16/content_9613.htm (2015).

43. Liu, S., Ni, J., Radcliffe, J.S. & Vonderohe, C.E. Mitigation of ammonia emissions from pig production using reduced dietary crude protein with amino acid supplementation. Bioresour. Technol. **233**, 200-208 (2017).

44. Ndegwa, P.M., Hristov, A.N., Arogo, J. & Sheffield, R.E. A review of ammonia emission mitigation techniques for concentrated animal feeding operations. Biosyst. Eng. **100**, 453-469 (2008).

45. Witzke, H.P. & Oenema, O. Assessment of Most Promising measures task 3 contract integrated measures in agriculture to reduce ammonia emission. http://www.scamonia.wur.nl. (2007).

46. Pope, T. & Emmert, J.L. Phase-feeding supports maximum growth performance of broiler chicks from forty-three to seventy-one days of age. Poultry Science **80**, 345-352 (2001).

47. MEP. Regulation on the Prevention and Control of Pollution from Breeding of Livestock and Poultry. in *[2010]151* (2010).

48. Wagner, S., Angenendt, E., Beletskaya, O. & Zeddies, J. Assessing ammonia emission abatement measures in agriculture: Farmers' costs and society's benefits - A case study for Lower Saxony, Germany. Agric. Syst. **157**, 70-80 (2017).

49. Chadwick, D., *et al*. Improving manure nutrient management towards sustainable agricultural intensification in China. Agr. Ecosyst. Environ. **209**, 34-46 (2015).

50. Zhang, C., *et al*. Rebuilding the linkage between livestock and cropland to mitigate agricultural pollution in China. Resour. Conser. Recy. **144**, 65-73 (2019).

51. Bai, Z., *et al*. Nitrogen, Phosphorus, and Potassium Flows through the Manure Management Chain in China. Environ. Sci. Technol. **50**, 13409-13418 (2016).

52. Liu, X. & Li, S. Temporal and spatial distribution of nutrient resource from livestock and poultry feces and its returning to cropland. Trans ASAE **34**, 1-14 (2018).

53. Li, Q., *et al*. Effect of a new urease inhibitor on ammonia volatilization and nitrogen utilization in wheat in north and northwest China. Field Crops Res. **175**, 96-105 (2015).

54. Central Committee of the Communist Party of China (CPC). The 13th five-year plan for economic and social development of the People's Republic of China (2016-2020). (2016).

55. MOA. National sustainable agricultural development plan (2015-2030).

http://jiuban.moa.gov.cn/zwllm/zcfg/qnhnzc/201505/t20150528_4622065.htm (2015).
56. Qixun. Market prospect analysis and forecast report of soil amendment in China in 2019-2024. http://www.qxcu.com/report/519/1344183.htm (2019).
57. Zhou, Y. & Wu, J. Research status, problems and prospects of soil amentment. Henan Agric. Sci., 152-155 (2010).
58. The State Council. The 13th Five-Year Plan (2016-2020) for the Protection of Ecological Environment. (2016).
59. Liubo, Z. Ammonia emission characteristics and mitigation solutions in dairy farming system in specific regions. (Agricultural University of Hebei, 2015).
60. Du, Y., *et al*. A global strategy to mitigate the environmental impact of China's ruminant consumption boom. Nat. Commun. **9** (2018).
61. Zhang, T., *et al*. Long-term manure application increased greenhouse gas emissions but had no effect on ammonia volatilization in a Northern China upland field. Sci. Total Environ. **633**, 230-239 (2018).
62. HUANG, Y. & TANG, Y. An estimate of greenhouse gas (N_2O and CO_2) mitigation potential under various scenarios of nitrogen use efficiency in Chinese croplands. Glob. Change. Biol. **16**, 2958-2970 (2010).
63. Xia, L., *et al*. Simultaneous quantification of N_2,NH_3 and N_2O emissions from a flooded paddy field under different N fertilization regimes. Glob. Change. Biol. **26**, 2292-2303 (2020).
64. Lu, W.F., *et al*. Methane Emissions and Mitigation Options in Irrigated Rice Fields in Southeast China. Nutr. Cycling Agroecosyst. **58**, 65-73 (2000).
65. Cao, Y., *et al*. Mitigation of ammonia, nitrous oxide and methane emissions during solid waste composting with different additives: A meta-analysis. J. Clean. Prod. **235**, 626-635 (2019).
66. Wang, Y., *et al*. Mitigating Greenhouse Gas and Ammonia Emissions from Beef Cattle Feedlot Production: A System Meta-Analysis. Environ. Sci. Technol. **52**, 11232-11242 (2018).
67. Wang, Y., *et al*. Mitigating Greenhouse Gas and Ammonia Emissions from Swine Manure Management: A System Analysis. Environ. Sci. Technol. **51**, 4503-4511 (2017).
68. Agyarko-Mintah, E., *et al*. Biochar increases nitrogen retention and lowers greenhouse gas emissions when added to composting poultry litter. Waste Manag. **61**, 138-149 (2017).
69. IPCC. Climate Change 2013: The Physical Science Basis. Contribution of Working Group I to the Fifth Assessment Report of the Intergovernmental Panel on Climate Change. www.climatechange2013.org (2013).
70. Ding, W., *et al*. Improving yield and nitrogen use efficiency through alternative fertilization options for rice in China: A meta-analysis. Field Crops Res. **227**, 11-18 (2018).
71. Liu, T., Huang, J., Chai, K., Cao, C. & Li, C. Effects of N Fertilizer Sources and Tillage Practices on NH_3 Volatilization, Grain Yield, and N Use Efficiency of Rice Fields in Central China. Front. Plant Sci. **9** (2018).
72. Win, K.T., Nonaka, R., Toyota, K., Motobayashi, T. & Hosomi, M. Effects of option mitigating ammonia volatilization on CH4 and N2O emissions from a paddy field fertilized with anaerobically digested cattle slurry. Biol. Fertil. Soils **46**, 589-595 (2010).
73. Rose, T.J., Wood, R.H., Rose, M.T. & Van Zwieten, L. A re-evaluation of the agronomic effectiveness of the nitrification inhibitors DCD and DMPP and the urease inhibitor NBPT. Agric. Ecosyst. Environ. **252**, 69-73 (2018).
74. Drury, C.F., *et al*. Combined Effects of N Fertilizer Placement and Enhanced Efficiency Fertilizers to

Reduce N Losses from Corn Production. https://fertilizercanada.ca/wp-content/uploads/2017/01/ASA-4R-Research-Presentations_Binder-1.pdf (2017).

75. Kawakami, E.M., Oosterhuis, D.M., Snider, J.L. & Mozaffari, M. Physiological and yield responses of field-grown cotton to application of urea with the urease inhibitor NBPT and the nitrification inhibitor DCD. Eur. J. Agron. **43**, 147-154 (2012).

76. Wu, J., *et al*. Greenhouse Gas Emissions from Cotton Field under Different Irrigation Methods and Fertilization Regimes in Arid Northwestern China. Sci. World J. **2014**, 1-10 (2014).

77. Freney, J.R., *et al*. Factors controlling ammonia loss from trash covered sugarcane fields fertilized with urea. Fertil. Res. **31**, 341-349 (1992).

78. Dattamudi, S., Wang, J.J., Dodla, S.K., Arceneaux, A. & Viator, H.P. Effect of nitrogen fertilization and residue management practices on ammonia emissions from subtropical sugarcane production. Atmos. Environ. **139**, 122-130 (2016).

79. Guo, Y., Li, B., Di, H., Zhang, L. & Gao, Z. Effects of dicyandiamide (DCD) on nitrate leaching, gaseous emissions of ammonia and nitrous oxide in a greenhouse vegetable production system in northern China. Soil Sci. Plant Nutr. **58**, 647-658 (2012).

80. Min, J., Zhao, X., Shi, W., Xing, G. & Zhu, Z. Nitrogen Balance and Loss in a Greenhouse Vegetable System in Southeastern China. Pedosphere **21**, 464-472 (2011).

81. Jia, J., Li, B., Chen, Z., Xie, Z. & Xiong, Z. Effects of biochar application on vegetable production and emissions of N_2O and CH_4. Soil Sci. Plant Nutr. **58**, 503-509 (2012).

82. Deng, J., Li, C. & Wang, Y. Modeling ammonia emissions from dairy production systems in the United States. Atmos. Environ. **114**, 8-18 (2015).

83. Zhang, N., *et al*. Reducing Ammonia Emissions from Dairy Cattle Production via Cost-Effective Manure Management Techniques in China. Environ. Sci. Technol. **53**, 11840-11848 (2019).

84. Poteko, J., Zähner, M. & Schrade, S. Effects of housing system, floor type and temperature on ammonia and methane emissions from dairy farming: A meta-analysis. Biosyst. Eng. **182**, 16-28 (2019).

85. Burchill, W., Reville, F., Misselbrook, T.H., O'Connell, C. & Lanigan, G.J. Ammonia emissions and mitigation from a concrete yard used by cattle. Biosyst. Eng. **184**, 181-189 (2019).

86. Marino, R., *et al*. Climate change: Production performance, health issues, greenhouse gas emissions and mitigation strategies in sheep and goat farming. Small Rumin. Res. **135**, 50-59 (2016).

87. Liu, H. & Zhou, D. Mitigation of ammonia and nitrous oxide emissions from pasture treated with urine of sheep fed diets supplemented with sodium chloride. Anim. Feed Sci. Technol. **192**, 39-47 (2014).

88. Silaban, R., *et al*. Nitrogen and Ammonia Mitigation on Laying Hen Farms: Effects of Low-protein Diet and Manure Filtering. Int. J. Poult. Sci. (2017).

89. Wang, Y., *et al*. Mitigating ammonia emissions from typical broiler and layer manure management – A system analysis. Waste Manag. **93**, 23-33 (2019).

90. Amber, K., Yakout, H.M. & Hamed, R.S. Effect of feeding diets containing yucca extract or probiotic on growth, digestibility, nitrogen balance and caecal microbial activity of growing New Zealand white rabbits. in *8th World Rabbit Congress* (Puebla, Mexico, 2004).

91. Liu, L. Ventilation and ammonia removal of rabbit house in winter. Jilin Anim. Husb. Vet. Med. **31**, 40 (2010).

92. Airaksinen, S., Heinonen-Tanski, H. & Heiskanen, M. Quality of different bedding materials and their

influence on the compostability of horse manure. J. Equine Vet. Sci. **21**, 125-130 (2001).
93. Hadin, Å., Eriksson, O. & Hillman, K. A review of potential critical factors in horse keeping for anaerobic digestion of horse manure. Renew. Sustain. Energy Rev. **65**, 432-442 (2016).
94. Li, X., Jin, H. & Xue, S. Current situation and prospect of camel breeding in China. Anim. Feed Sci. Technol. **28**, 72-74 (2007).
95. Zhang, Q., Gao, S. & Wang, G. A brief analysis of the development direction of camel breeding. China animal husbandry and veterinary abstract **34**, 87, 231 (2018).
96. The World Bank. The World Bank Data. https://data.worldbank.org/ (2019).
97. IFADATA. International Fertilizer Industry Association (2018). http://ifadata.fertilizer.org/ucSearch.aspx (2018).
98. NBSC. China Rural Statistics Yearbook（1985-2018）. (China Statistics Press, 2019).
99. Chinese Nutrition Society. *Dietary guidelines for Chinese residents (2016)* (People's Medical Publishing House, Beijing, 2016).
100. NBSC. National Bureau of Statistics of China. China Statistical Yearbook (2000-2018). (China Statistics Press, 2019).
101. EEA. European Union emission inventory report 1990-2017. https://www.eea.europa.eu/publications/european-union-emissions-inventory-report-2017 (2019).
102. The Ministry of Agriculture (MOA). China Agriculture Outlook Report (2018-2027). (2018).
103. Clean Air Alliance of China (CAAS). Air pollution prevention and control action plan. (2013).
104. The State Council. National Agricultural Water-Saving Outline (2012-2020). http://www.gov.cn/zwgk/2012-12/15/content_2291002.htm (2012).
105. Finzi, A., *et al*. Comparison of techniques for ammonia emission mitigation during storage of livestock manure and assessment of their effect in the management chain. J. Agric. Eng. **50**, 12-19 (2019).
106. Sanz-Cobena, A., Misselbrook, T., Camp, V. & Vallejo, A. Effect of water addition and the urease inhibitor NBPT on the abatement of ammonia emission from surface applied urea. Atmos. Environ. **45**, 1517-1524 (2011).
107. McCubbin, D.R., Apelberg, B.J., Roe, S. & Divita, F. Livestock Ammonia Management and Particulate-Related Health Benefits. Environ. Sci. Technol. **36**, 1141-1146 (2002).
108. FAOSTAT. Food and Agriculture Organization of the United Nations Online Statistical Databases. http://www.fao.org/faostat/en/#data (2018).
109. Ma, L., *et al*. Exploring Future Food Provision Scenarios for China. Environ. Sci. Technol. **53**, 1385-1393 (2018).
110. MOA. Ministry of Agriculture. Zero Increase Action Plan on Fertilizer Use by 2020. http://jiuban.moa.gov.cn/zwllm/tzgg/tz/201503/t20150318_4444765.htm (2015).
111. Sheng, Y. & Song, L. Agricultural production and food consumption in China: A long-term projection. China Econ. Rev. **53**, 15-29 (2019).
112. Zhai, Z., Chen, J. & Li, L. Future Trends of China's Population and Aging: 2015~2100. Popul. Res., 60-71 (2017).
113. The State Council of China. National population development plan (2016-2030). (2016).
114. The State Council. National plan for Agricultural Modernization (2016-2020). http://www.gov.cn/zhengce/content/2016-10/20/content_5122217.htm (2016).

115. Alexandratos, N. & Bruinsma, J. World agriculture towards 2030/2050: the 2012 revision. (2012).
116. IIASA. The online version of the GAINS Model. http://www.iiasa.ac.at/ (2019).
117. Yang, G. Population Expectations and Policy Prospects After the Universal Two-child Policy. **16**, 25-33 (2016).
118. IPCC. IPCC Data distribution Centre. https://www.ipcc-data.org/guidelines/index.html (2019).
119. Bai, Z., *et al*. China's livestock transition: Driving forces, impacts, and consequences. Sci. Adv. **4**, r8534 (2018).
120. Gu, B., Ju, X., Chang, J., Ge, Y. & Vitousek, P.M. Integrated reactive nitrogen budgets and future trends in China. Proc. Natl Acad. Sci. USA **112**, 8792-8797 (2015).
121. Wang, G., *et al*. Persistent sulfate formation from London Fog to Chinese haze. Proc. Natl Acad. Sci. USA, 201616540 (2016).
122. Liu, M., *et al*. Ammonia emission control in China would mitigate haze pollution and nitrogen deposition but worsen acid rain. Proc. Natl Acad. Sci. USA, 201814880 (2019).
123. Xu, Z., *et al*. High efficiency of livestock ammonia emission controls in alleviating particulate nitrate during a severe winter haze episode in northern China. Atmos. Chem. Phys. **19**, 5605-5613 (2019).
