# Supplementary material for: Societal benefits of halving agricultural ammonia emissions in China far exceed the abatement costs
Source: Nat Commun. 2020 Aug 31;11:4357. doi: 10.1038/s41467-020-18196-z (PMC7459339; doi:10.1038/s41467-020-18196-z)
Supplement: Supplementary file 3 — Reporting Summary [file 41467_2020_18196_MOESM3_ESM.pdf]

nature research

Corresponding author(s): Baojing Gu & Deli Chen

Last updated by author(s): Jul 25, 2020

# Reporting Summary

Nature Research wishes to improve the reproducibility of the work that we publish. This form provides structure for consistency and transparency in reporting. For further information on Nature Research policies, see Authors & Referees and the Editorial Policy Checklist.

## Statistics

For all statistical analyses, confirm that the following items are present in the figure legend, table legend, main text, or Methods section.

| n/a | Confirmed | |
|---|---|---|
| ☒ | ☐ | The exact sample size (*n*) for each experimental group/condition, given as a discrete number and unit of measurement |
| ☒ | ☐ | A statement on whether measurements were taken from distinct samples or whether the same sample was measured repeatedly |
| ☒ | ☐ | The statistical test(s) used AND whether they are one- or two-sided<br>*Only common tests should be described solely by name; describe more complex techniques in the Methods section.* |
| ☒ | ☐ | A description of all covariates tested |
| ☐ | ☒ | A description of any assumptions or corrections, such as tests of normality and adjustment for multiple comparisons |
| ☐ | ☒ | A full description of the statistical parameters including central tendency (e.g. means) or other basic estimates (e.g. regression coefficient) AND variation (e.g. standard deviation) or associated estimates of uncertainty (e.g. confidence intervals) |
| ☒ | ☐ | For null hypothesis testing, the test statistic (e.g. *F*, *t*, *r*) with confidence intervals, effect sizes, degrees of freedom and *P* value noted<br>*Give P values as exact values whenever suitable.* |
| ☒ | ☐ | For Bayesian analysis, information on the choice of priors and Markov chain Monte Carlo settings |
| ☒ | ☐ | For hierarchical and complex designs, identification of the appropriate level for tests and full reporting of outcomes |
| ☒ | ☐ | Estimates of effect sizes (e.g. Cohen's *d*, Pearson's *r*), indicating how they were calculated |

*Our web collection on statistics for biologists contains articles on many of the points above.*

## Software and code

Policy information about availability of computer code

| | |
|---|---|
| Data collection | No software was used. |
| Data analysis | Data analysis in this study are conducted at Microsoft Excel and CHANS model. Simplified CHANS calculator could be founded at https://person.zju.edu.cn/en/bjgu#930811. |

For manuscripts utilizing custom algorithms or software that are central to the research but not yet described in published literature, software must be made available to editors/reviewers. We strongly encourage code deposition in a community repository (e.g. GitHub). See the Nature Research guidelines for submitting code & software for further information.

## Data

Policy information about availability of data

All manuscripts must include a data availability statement. This statement should provide the following information, where applicable:

- Accession codes, unique identifiers, or web links for publicly available datasets
- A list of figures that have associated raw data
- A description of any restrictions on data availability

Data supporting the findings of this study is available within the article and its Supplementary Information files, or are available from the corresponding author upon reasonable request.

# Field-specific reporting

Please select the one below that is the best fit for your research. If you are not sure, read the appropriate sections before making your selection.

☐ Life sciences ☐ Behavioural & social sciences ☒ Ecological, evolutionary & environmental sciences

For a reference copy of the document with all sections, see nature.com/documents/nr-reporting-summary-flat.pdf

# Ecological, evolutionary & environmental sciences study design

All studies must disclose on these points even when the disclosure is negative.

| | |
|---|---|
| Study description | This study performed a comprehensive assessment of the NH3 mitigation potential, implementation costs and the associated societal benefits in China by integrating multiple methods for simulation of different mitigation pathways during 2020-2050. |
| Research sample | A total of 27 technical NH3 mitigation options are selected for 10 main crops and 10 animal types in China. |
| Sampling strategy | To identify the feasible and cost-effective NH3 abatement measures for Chinese agriculture, we reviewed all currently available mitigation options in China and other countries. Our criteria for the selection of abatement measures are: 1) mitigation efficiency, 2) implementation cost, 3) practical applicability 4) limitations, and 5) presence of co-benefits". The detailed selection criteria is listed in the "Methods" |
| Data collection | All data used in this study were collected from publicly available sources including literatures, open data websites (FAO,NBSC), government agencies websites(NBSC, MOA, EEA), and models(GAINS, WRF-CMAQ, CHANS) by the great majority of co-authors. |
| Timing and spatial scale | We conducted the integrated assessment of agricultural NH3 mitigation on a national scale. The time scale of data collected and projected in this study ranges from 2000 to 2050. |
| Data exclusions | No data were excluded from the analyses. |
| Reproducibility | All attempts to repeat the results of this study were successful. |
| Randomization | This is not applicable in this study because this study is mainly based on meta-analysis results. |
| Blinding | Blinding is not applicable in this study it is not a experiment-based study. |

Did the study involve field work? ☐ Yes ☒ No

# Reporting for specific materials, systems and methods

We require information from authors about some types of materials, experimental systems and methods used in many studies. Here, indicate whether each material, system or method listed is relevant to your study. If you are not sure if a list item applies to your research, read the appropriate section before selecting a response.

## Materials & experimental systems

| n/a | Involved in the study | |
|---|---|---|
| ☒ | ☐ | Antibodies |
| ☒ | ☐ | Eukaryotic cell lines |
| ☒ | ☐ | Palaeontology |
| ☒ | ☐ | Animals and other organisms |
| ☒ | ☐ | Human research participants |
| ☒ | ☐ | Clinical data |

## Methods

| n/a | Involved in the study | |
|---|---|---|
| ☒ | ☐ | ChIP-seq |
| ☒ | ☐ | Flow cytometry |
| ☒ | ☐ | MRI-based neuroimaging |
